# Phytochemical Investigation of Marker Compounds from Indigenous Korean *Salix* Species and Their Antimicrobial Effects

**DOI:** 10.3390/plants12010104

**Published:** 2022-12-26

**Authors:** Yoon Seo Jang, Da Eun Lee, Joo-Hyun Hong, Kyung Ah Kim, Bora Kim, Yeo Rang Cho, Moon-Jin Ra, Sang-Mi Jung, Jeong-Nam Yu, Seongpil An, Ki Hyun Kim

**Affiliations:** 1 School of Pharmacy, Sungkyunkwan University, Suwon 16419, Republic of Korea; 2 Hongcheon Institute of Medicinal Herb, Hongcheon-gun 25142, Republic of Korea; 3 Nakdonggang National Institute of Biological Resources, Sangju 37242, Republic of Korea; 4 SKKU Advanced Institute of Nanotechnology (SAINT), Sungkyunkwan University (SKKU), Suwon 16419, Republic of Korea; 5 Department of Nano Engineering, Sungkyunkwan University (SKKU), Suwon 16419, Republic of Korea

**Keywords:** *Salix* species, marker compound, LC/MS, antimicrobial effects

## Abstract

*Salix* species, including willow trees, are distributed in the temperate regions of Asian countries, including South Korea. Willow trees are used to treat pain and inflammatory diseases. Due to the medicinal properties of willow trees, pharmacological studies of other *Salix* spp. have gained attention; however, only a few studies have investigated the phytochemicals of these species. As part of our ongoing natural product research to identify bioactive phytochemicals and elucidate their chemical structures from natural resources, we investigated the marker compounds from indigenous Korean *Salix* species, namely, *Salix triandra*, *S. chaenomeloides*, *S. gracilistyla*, *S. koriyanagi*, *S. koreensis*, *S. pseudolasiogyne*, *S. caprea*, and *S. rorida*. The ethanolic extract of each *Salix* sp. was investigated using high-performance liquid chromatography combined with thin-layer chromatography and liquid chromatography–mass spectrometry-based analysis, and marker compounds of each *Salix* sp. were isolated. The chemical structures of the marker compounds (**1–8**), 3-(4-hydroxyphenyl)propyl β-D-glucopyranoside (**1**), 2-*O*-acetylsalicin (**2**), 1-*O*-*p*-coumaroyl glucoside (**3**), picein (**4**), isograndidentatin B (**5**), 2′-*O*-acetylsalicortin (**6**), dihydromyricetin (**7**), and salicin (**8**) were elucidated via nuclear magnetic resonance spectroscopy and high-resolution liquid chromatography–mass spectrometry using ultrahigh-performance liquid chromatography coupled with a G6545B Q-TOF MS system with a dual electrospray ionization source. The identified marker compounds **1–8** were examined for their antimicrobial effects against plant pathogenic fungi and bacteria. Dihydromyricetin (**7**) exhibited antibacterial activity against *Staphylococcus aureus*, inducing 32.4% inhibition at a final concentration of 125 μg/mL with an MIC_50_ value of 250 μg/mL. Overall, this study isolated the marker compounds of *S. triandra*, *S. chaenomeloides*, *S. gracilistyla*, *S. koriyanagi*, *S. koreensis*, *S. pseudolasiogyne*, *S. caprea*, and *S. rorida* and identified the anti-*Staphylococcus aureus* bacterial compound dihydromyricetin.

## 1. Introduction

The genus *Salix* comprises approximately 500 species of deciduous trees and shrubs distributed in the temperate regions of Asian countries, including South Korea, and willow trees are the most representative plants of this genus [1]. Willow trees have been used for the treatment of pain and inflammatory diseases as their barks possess anti-inflammatory metabolites, such as salicylic acid, which is useful as a natural source of aspirin [2,3]. Owing to the use of willow trees for medicinal purposes, pharmacological studies of *Salix* spp. have gained attention, and many studies have demonstrated their biological and beneficial effects, including anti-inflammatory, antitumor, antioxidant, and antiobesity effects [4,5,6,7]. Specifically, salicin derivatives identified from *Salix* spp. exert anti-inflammatory effects by inhibiting lipopolysaccharide (LPS)-induced nitric oxide (NO) production in BV2 microglial cells [8].

*Salix triandra*, also known as almond willow, is native to Western and Central Asia and Europe. It is also used as a basket-making material. A pharmacological study of *S. triandra* reported that *S. triandra* leaf extract showed antioxidant activity by promoting the removal of 1,1-diphenyl-2-picrylhydrazyl (DPPH) [9]. Despite its health benefits, only a few studies have been conducted on the chemical constituents of *S. triandra*. *S. chaenomeloides* (also known as *S. glandulosa*), commonly called pussy willow, an indigenous plant found in East Asia. A previous phytochemical investigation of *S. chaenomeloides* reported the presence of phenolic glycosides and salicin derivatives [10,11], which showed inhibitory effects on NO production and anti-neuroinflammatory effects in LPS-activated murine microglial cells. *S. gracilistyla* is distributed along river coasts. Its stems and leaves are used in traditional medicine to treat skin diseases, wounds, and rooting arthritis, and its bark is used as a painkiller [12]. A pharmacological study of *S. gracilistyla* reported that the *S*. *gracilistyla* extract showed anti-inflammatory activity by inhibiting NO production in LPS-activated macrophages [13]. In addition, *S. gracilistyla* extracts can be used as effective cosmetic ingredients due to their antioxidant and skin-whitening activities [14]. However, only a few studies have been conducted on these phytochemicals. *S. koriyanagi* is found in fertile moist soil near rivers, streams, and valleys, and is generally called winnow willow, implying that the bark of this tree can be used to make winnowing baskets and traditional crafts [15]. *S. koriyanagi* is an endemic Korean species owing to its isolated distribution and high conservation priority [16], and few studies have investigated its pharmacological activities and phytochemicals. *S. koreensis*, also known as the Korean willow, grows along most rivers in Korea [17]. *S. koreensis* is traditionally used for brushing teeth after meals to clean the oral cavity and prevent oral inflammation and tooth decay [18]. A pharmacological study of *S. koreensis* reported that the *S. koreensis* extract showed anticancer activity by inhibiting cell growth and promoting apoptosis in human colon and lung cancer cells [19]. *S. koreensis* extract also showed antioxidant, anti-inflammatory, and hepatoprotective effects [20,21,22]. Despite its diverse pharmacological effects, only a few studies have investigated the chemical constituents of *S. koreensis*. *S. pseudolasiogyne*, also known as the weeping willow, is mostly found in Asian countries, including South Korea. It is used as a traditional Korean medicine for the treatment of pain and fever [23] Recent pharmacological studies have revealed that extracts of *S. pseudolasiogyne* twigs exert antiadipogenic and anti-amnesic effects [24,25]. Previous phytochemical investigations revealed salicin as the primary constituent of *S. pseudolasiogyne* twigs and reported salicin derivatives, such as 2′-*O*-acetylsalicin, salicortin, 2′-*O*-acetylsalicortin, 3′-*O*-acetylsalicortin, and 6′-*O*-acetylsalicortin, in *S. pseudolasiogyne* extracts [8,24]. *S. caprea*, also known as the goat willow, is mostly found in European and Asian countries, including South Korea. It is used in traditional Korean medicine for the treatment of pain and fever. Recent pharmacological studies have revealed that the EtOH extract of *S. caprea* flowers shows antioxidant activity to dose-dependently scavenge DPPH, superoxide (O2·−), and hydrogen peroxide (H_2_O_2_) [6] and that *S. caprea* extract inhibits 7,12-dimethyl benz[a] anthracene-induced phorbol ester-promoted skin carcinogenesis [4]. However, only a few phytochemical studies have focused on *S. caprea*. Finally, *S. rorida* is a deciduous tree and a willow species native to Japan, northern China, Korea, and Russia. A recent phytochemical study revealed salicin as the major constituent of *S. rorida* and identified other constituents, including (+)-catechin, naringenin, salipurposide, aromadendrin, isosalipurposide, aromadendrin-7-*O*-β-D-glucopyranoside, and taxifolin-7-*O*-β-D-glucopyranoside, via liquid chromatography–mass spectrometry (LC/MS) analysis [25]. However, the pharmacological activity of *S. rorida* has not yet been elucidated.

As part of our ongoing research projects to identify bioactive natural products and elucidate their chemical structures from natural resources [26,27,28,29,30,31,32], we investigated the marker compounds from the Korean indigenous *Salix* species *S. triandra*, *S. chaenomeloides*, *S. gracilistyla*, *S. koriyanagi*, *S. koreensis*, *S. pseudolasiogyne*, *S. caprea*, and *S. rorida*. Ethanolic extracts of these *Salix* spp. were investigated using high-performance liquid chromatography (HPLC) combined with thin-layer chromatography (TLC) and LC/MS-based analysis, followed by the isolation of each marker compound of *Salix* spp. The chemical structures of marker compounds (**1**–**8**) were elucidated via nuclear magnetic resonance (NMR) spectroscopy and electrospray ionization (ESI) LC/MS analyses. Finally, the identified marker compounds **1**–**8** were tested for their antimicrobial effects. Herein, we report the isolation and structural characterization of marker compounds **1**–**8** as well as their bioactivity with respect to their antimicrobial effects.

## 2. Results and Discussion

### 2.1. Collection of Salix Species

**Figure 1:**
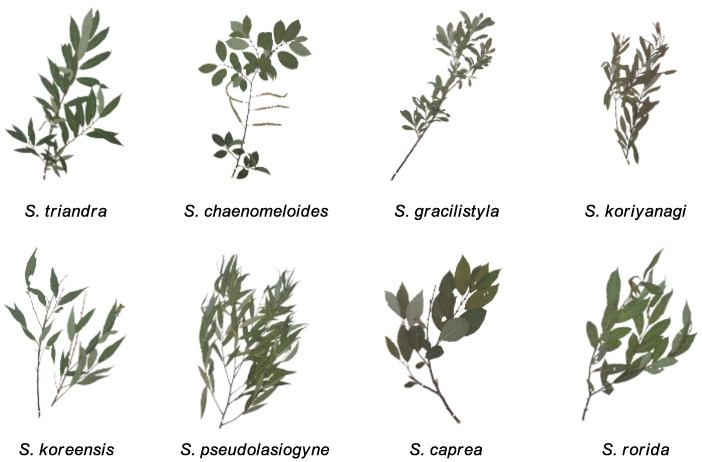
Specimens of the eight collected *Salix* species (*S*. *triandra*, *S. chaenomeloides*, *S. gracilistyla*, *S. koriyanagi*, *S. koreensis*, *S. pseudolasiogyne*, *S. caprea*, and *S. rorida*).

The twigs of eight *Salix* species (*S*. *triandra*, *S. chaenomeloides*, *S. gracilistyla*, *S. koriyanagi*, *S. koreensis*, *S. pseudolasiogyne*, *S. caprea*, and *S. rorida*) were collected from Chungcheongnam-do, Chungcheongbuk-do, and Gangwon-do (South Korea), thoroughly dried, and mounted. Plant specimens (Figure 1) were made into herbarium vouchers and stored at room temperature in the dark. Twigs of the collected *Salix* species were cut into small pieces and extracted with 80% ethanol (EtOH) to obtain EtOH crude extracts.

### 2.2. Isolation and Identification of Marker Compounds

**Figure 2:**
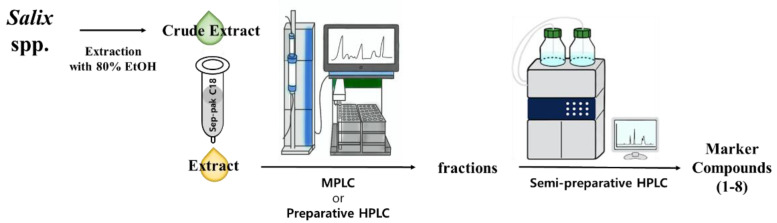
Separation scheme of the marker compounds **1**–**8**.

**Figure 3:**
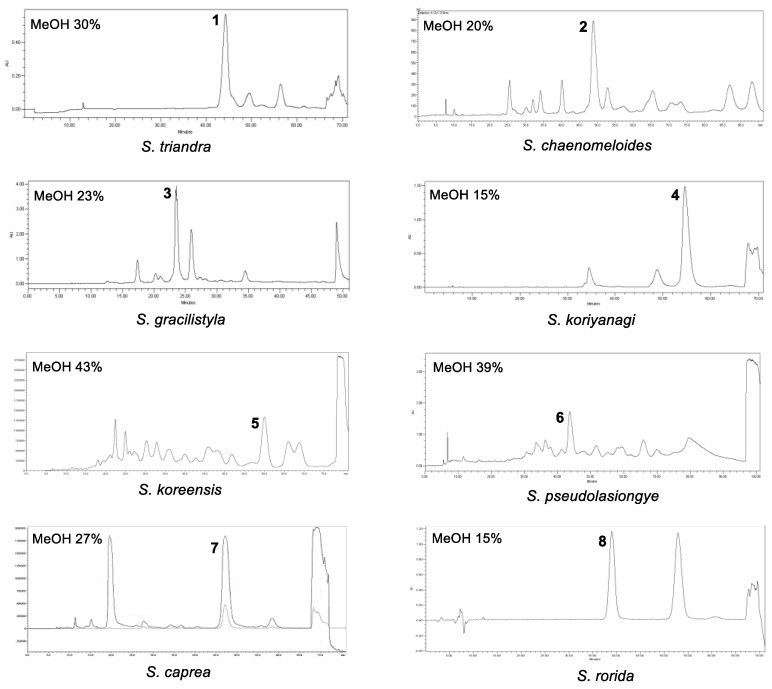
HPLC chromatogram for isolation of marker compounds **1–8** from the eight collected *Salix* species.

Marker compounds are the pure or single isolated chemical constituents within a crude plant extract or medicinal herbal drug that can confirm the exact botanical identity of material. Marker compounds are of interest for quality control purposes; however, marker compounds may or may not contribute to the therapeutic activity of crude extracts or herbal drugs. Marker compounds can serve as active principals of the herbal drug in the preparation or the finished product. Marker compounds are applied at various stages in the development and manufacturing of herbal medicine, including authentication and differentiation of species, quality evaluation and stability assessment, and collecting and harvesting. LC/MS-based analysis combined with our in-house UV library and TLC analysis of crude EtOH extracts from the eight *Salix* species (*S. triandra*, *S. chaenomeloides*, *S. gracilistyla*, *S. koriyanagi*, *S. koreensis*, *S. pseudolasiogyne*, *S. caprea*, and *S. rorida*) facilitated the determination of the marker compounds for these species based on the amount of the component present in each extract. Phytochemical investigation of the crude EtOH extracts was performed via TLC and LC/MS-based analysis using column chromatography and HPLC to isolate the marker compounds (Figure 2). Final semipreparative HPLC separation revealed each marker compound from the fraction via LC/MS analysis, where 3-(4-hydroxyphenyl)propyl β-D-glucopyranoside (**1**) was isolated from *S. triandra*, 2-*O*-acetylsalicin (**2**) from *S. chaenomeloides*, 1-*O*-*p*-coumaroyl glucoside (**3**) from *S. gracilistyla*, picein (**4**) from *S. koriyanagi*, isograndidentatin B (**5**) from *S. koreensis*, 2′-*O*-acetylsalicortin (**6**) from *S. pseudolasiogyne*, dihydromyricetin (**7**) from *S. caprea*, and salicin (**8**) from *S. rorida* (Figure 3).

The marker compounds of *Salix* spp. were structurally elucidated as 3-(4-hydroxyphenyl)propyl β-D-glucopyranoside (**1**) [33], 2-*O*-acetylsalicin (**2**) [34], 1-*O*-*p*-coumaroyl glucoside (**3**) [35], picein (**4**) [36], isograndidentatin B (**5**) [37], 2′-*O*-acetylsalicortin (**6**) [38], dihydromyricetin (**7**) [39], and salicin (**8**) [40] (Figure 4) via NMR spectroscopy and high-resolution (HR)-ESI MS analysis (Figures S1–S16) using Agilent 1290 Infinity II ultrahigh-performance liquid chromatography coupled with a G6545B Q-TOF MS system with a dual ESI source.

### 2.3. Evaluation of the Antimicrobial Activities of the Marker Compounds

Plant diseases caused by fungi, bacteria, and viruses can cause significant damage to the yield and quality of crops, fruit, and vegetables. Synthetic pesticides are widely used to control plant diseases in agriculture. However, adverse effects of chronic exposure to synthetic chemicals and concerns about environmental pollution and pesticide resistance have prompted the need for the development of new ecofriendly plant protection agents [41,42]. Thus, the antifungal activities of all isolated marker compounds were evaluated against the plant pathogenic fungi, *Fusarium solani*, *F. asiaticum*, *Botrytis cinerea*, *Cylindrocarpon destructans*, and *Rhizoctonia solani.* Antifungal activity tests revealed that all tested compounds were inactive (data not shown). Next, the marker compounds were tested for their antibacterial activity against the Gram-positive bacterium *Staphylococcus aureus* (HG003) and Gram-negative bacterium *Escherichia coli* (MG1655). Among the isolates, only dihydromyricetin (**7**) exhibited antibacterial activity against *S. aureus*, inducing 32.4% inhibition at a final concentration of 125 μg/mL with an MIC_50_ value of 250 μg/mL (Table 1). Meanwhile, 3-(4-hydroxyphenyl)propyl β-D-glucopyranoside (**1**) and 1-*O*-*p*-coumaroyl glucoside (**3**) exhibited very weak activity against *S. aureus*, inducing only 1.9% and 1.3% inhibition, respectively (Table 1), and the other compounds had no effects on the growth of *S. aureus* and *E. coli*.

## 3. Materials and Methods

### 3.1. General Experimental Procedure

The experimental procedure is described in detail in Supplementary Materials.

### 3.2. Plant Materials

In June 2021, twigs of *S*. *triandra* were collected from Yesan-gun and those of *S. pseudolasiogyne* from Asan-si (Chungcheongnam-do, South Korea). Twigs of *S. chaenomeloides*, *S. koriyanagi*, and *S. caprea* were collected from Inje-gun and those of *S. rorida* from Hwacheon-gun (Gangwon-do, South Korea). The twigs of *S. gracilistyla* and *S. koreensis* were collected from Jecheon-si (Chungcheongbuk-do, South Korea). Each material was authenticated by the author J.N.Yu. Voucher specimens of the materials (HIMH-2104 for *S*. *triandra*, HIMH-2105 for *S. chaenomeloides*, HIMH-2106 for *S. gracilistyla*, HIMH-2107 for *S. koriyanagi*, HIMH-2108 for *S. koreensis*, HIMH-2109 for *S. pseudolasiogyne*, HIMH-2110 for *S. caprea*, and HIMH-2111 for *S. rorida*) were deposited at the Hongcheon Institute of Medicinal Herbs, Hongcheon-gun, South Korea.

### 3.3. Extraction and Isolation of Marker Compounds

#### 3.3.1. *S*. *triandra*

*S. triandra* twigs (1.6 kg) were dried at 35–45 °C in a plant-drying oven for one week, pulverized, and extracted with 80% ethanol (10 L) via sonication for 90 min three times at room temperature. The filtered ethanol extract was evaporated in vacuo to obtain the crude ethanol extract (86.6 g). The extract (10.1 g) was dissolved in MeOH (100 mL) and applied to a reverse-phase (RP) Sep-Pak column with 100% MeOH to remove the wax, lipids, and fatty acids, and the resultant residue was concentrated using an evaporator to obtain the crude extract (5.2 g). The crude extract (1.0 g) was separated via preparative RP-HPLC (from 30 to 80% MeOH for 80 min, gradient system) to obtain four fractions (P1–P4). Fraction P2 (303.3 mg) was isolated via semipreparative RP-HPLC using 30% MeOH to obtain marker compound **1** (9.6 mg, *t*_R_ = 44.0 min, ESI-MS [negative-ion mode] *m/z* 313 [M-H]^−^).

#### 3.3.2. *S. chaenomeloides*

*S. chaenomeloides* twigs (1.8 kg) were dried at 35–45 °C in a plant-drying oven for one week, pulverized, and extracted with 80% ethanol (10 L) via sonication for 90 min three times at room temperature. The filtered ethanol extract was evaporated in vacuo to obtain the crude ethanol extract (144.6 g). The extract (10.3 g) was dissolved in MeOH (100 mL) and applied to an RP Sep-Pak column with 100% MeOH to remove the wax, lipids, and fatty acids, and the resultant residue was concentrated using an evaporator to obtain the crude extract (7.2 g). The crude extract (1.0 g) was separated via preparative RP-HPLC (from 20 to 30% MeOH for 80 min, gradient system) to obtain five fractions (P1–P5). Fraction P4 (68.2 mg) was isolated via semipreparative RP-HPLC using 20% MeOH to obtain marker compound **2** (10.3 mg, *t*_R_ = 48.0 min, ESI-MS [positive-ion mode] *m/z* 329 [M + H]^+^).

#### 3.3.3. *S. gracilistyla*

*S. gracilistyla* twigs (1.0 kg) were dried at 35–45 °C in a plant-drying oven for one week, pulverized, and extracted with 80% ethanol (10 L) via sonication for 90 min three times at room temperature. The filtered ethanol extract was evaporated in vacuo to obtain the crude ethanol extract (163 g). The extract (11 g) was dissolved in MeOH (100 mL) and applied to an RP Sep-Pak column with 100% MeOH to remove the wax, lipids, and fatty acids, and the resultant residue was concentrated using an evaporator to obtain the crude extract (5.6 g). The crude extract (1.0 g) was separated via preparative RP-HPLC (from 20 to 30% MeOH for 80 min, gradient system) to obtain five fractions (P1–P5). Fraction P4 (468 mg) was isolated via semipreparative RP-HPLC using 23% MeOH to obtain marker compound **3** (9.8 mg, *t_R_* = 23.0 min, ESI-MS [negative-ion mode] *m/z* 325 [M-H]^−^).

#### 3.3.4. *S. koriyanagi*

*S. koriyanagi* twigs (2.0 kg) were dried at 35–45 °C in a plant-drying oven for one week, pulverized, and extracted with 80% ethanol (10 L) via sonication for 90 min three times at room temperature. The filtered ethanol extract was evaporated in vacuo to obtain the crude ethanol extract (167 g). The extract (10 g) was dissolved in MeOH (100 mL) and applied to an RP Sep-Pak column with 100% MeOH to remove the wax, lipids, and fatty acids, and the resultant residue was concentrated using an evaporator to obtain the crude extract (6.5 g). The crude extract (6.5 g) was subjected to HP-20 column chromatography, with eluting solvents of distilled water, MeOH, and acetone, to obtain three fractions (P1–P3). Fraction P1 (2.6 g) was separated via preparative RP-HPLC (from 20 to 30% MeOH for 82 min, gradient system) to obtain five fractions (P11–P15). Fraction P13 (245 mg) was isolated via semipreparative RP-HPLC using 15% MeOH to obtain marker compound **4** (10.5 mg, *t_R_* = 54.0 min, ESI-MS [negative-ion mode] *m/z* 297 [M-H]^−^).

#### 3.3.5. *S. koreensis*

*S. koreensis* twigs (2.3 kg) were dried at 35–45 °C in a plant-drying oven for one week, pulverized, and extracted with 80% ethanol (10 L) via sonication for 90 min three times at room temperature. The filtered ethanol extract was evaporated in vacuo to obtain the crude ethanol extract (101 g). The extract (9.1 g) was dissolved in MeOH (100 mL) and applied to an RP Sep-Pak column using 100% MeOH to remove the wax, lipids, and fatty acids, and the resultant residue was concentrated using an evaporator to obtain the crude extract (7.9 g). The crude extract (7.9 g) was subjected to HP-20 column chromatography, with the eluting solvents distilled water, MeOH, and acetone to obtain three fractions (P1–P3). Fraction P3 (582 mg) was separated via preparative RP-HPLC (from 20% to 100% MeOH for 101 min, gradient system) to obtain four fractions (P31–P34). Fraction P33 (582 mg) was isolated via semipreparative RP-HPLC using 43% MeOH to obtain marker compound **5** (11.5 mg, *t_R_* = 60.0 min, ESI-MS [positive-ion mode] *m/z* 447 [M + Na]^+^).

#### 3.3.6. *S. pseudolasiogyne*

*S. pseudolasiogyne* twigs (1.5 kg) were dried at 35–45 °C in a plant-drying oven for one week, pulverized, and extracted with 80% ethanol (10 L) via sonication for 90 min three times at room temperature. The filtered ethanol extract was evaporated in vacuo to obtain the crude ethanol extract (123.6 g). The extract (9.1 g) was dissolved in MeOH (100 mL) and applied to an RP Sep-Pak column using 100% MeOH to remove the wax, lipids, and fatty acids, and the resultant residue was concentrated using an evaporator to obtain the crude extract (4.0 g). The crude extract (4.0 g) was separated via preparative RP-HPLC (from 30 to 50% MeOH for 81 min, gradient system) to obtain four fractions (P1–P4). Fraction P4 (306 mg) was isolated via semipreparative RP-HPLC using 39% MeOH to obtain marker compound **6** (11.2 mg, *t_R_* = 44.0 min, ESI-MS [positive-ion mode] *m/z* 467 [M + H]^+^).

#### 3.3.7. *S. caprea*

*S. caprea* twigs (1.5 kg) were dried at 35–45℃ in a plant-drying oven for one week, pulverized, and extracted with 80% ethanol (10 L) via sonication for 90 min three times at room temperature. The filtered ethanol extract was evaporated in vacuo to obtain the crude ethanol extract (110.8 g). The crude extract (10.3 g) was separated using medium-pressure liquid chromatography (MPLC) (5%→10%→30%→100% MeOH, 92 min, gradient system) to yield seven fractions (P1–P7). Fraction P4 (507 mg) was isolated via semipreparative RP-HPLC using 27% MeOH to obtain marker compound **7** (11.8 mg, *t_R_* = 45.0 min, ESI-MS [positive-ion mode] *m/z* 321 [M + H]^+^).

#### 3.3.8. *S. rorida*

*S. rorida* twigs (1.5 kg) were dried at 35–45 °C in a plant-drying oven for one week, pulverized, and extracted with 80% ethanol (10 L) via sonication for 90 min three times at room temperature. The filtered ethanol extract was evaporated in vacuo to obtain the crude ethanol extract (143.3 g). The extract (10.5 g) was dissolved in MeOH (100 mL) and applied to an RP Sep-Pak column with 100% MeOH to remove the wax, lipids, and fatty acids, and the resultant residue was concentrated using an evaporator to obtain the crude extract (7.8 g). The crude extract (7.8 g) was separated by MPLC (from 0% to 50% MeOH for 70 min, gradient system) to obtain four fractions (P1–P4). Fraction P2 (218 mg) was isolated via semipreparative RP-HPLC using 15% MeOH to obtain marker compound **8** (15.2 mg, *t_R_* = 38.0 min, ESI-MS [positive-ion mode] *m/z* 304 [M + NH_4_]^+^).

### 3.4. Antifungal Activity

Five plant pathogenic fungi provided by the Korean Agricultural Culture Collection (KACC; Jeonju, South Korea). *B. cinerea* (KACC40965), *C. destructans* (KACC 41077), *F. solani* (KACC 44891), *F. asiaticum* (KACC 46429), and *R. solani* (KACC 48921), were used for the in vitro antifungal activity assay. Fungi were cultivated on 25 mL of malt extract agar (Difco, MD, USA) at 25 °C for 14 d in the dark. Marker compounds (**1**–**8**) were evaluated for their antifungal activities in a 96-well microplate [42]. Each well of the 96-well microplate contained 2 μL of the compound, 20 μL of spore suspension, and 178 μL of medium. Chemical fungicide benomyl was used as a positive control and dimethyl sulfoxide (DMSO; 1%) was used as a negative control for the antifungal assay. The plant pathogenic fungal spore suspension was adjusted to a density of 4 × 10^6^ cells/mL. The absorbance at 600 nm was measured every 12 h for 96 h. The experiments were performed in triplicate. The percentage of growth inhibition (GI%) was estimated using the following formula: GI% = 100 (A_control_ − A_test sample_)/(A_control_).

### 3.5. Antibacterial Activity

For the antibacterial activity assay, the Gram-positive bacterium *S. aureus* (HG033) and the Gram-negative bacterium *E. coli* (MG1655) were used. *Staphylococcus aureus* strains were maintained aerobically in tryptic soy broth (TSB, BD) or TSB 1.5% agar at 30 °C with shaking at 250 rpm. *E. coli* strains were maintained aerobically in lysogeny broth (LB, BD) or LB 1.5% agar at 37 °C with shaking at 250 rpm. The qualitative antibacterial activities of the marker compounds (**1**–**8**) were examined using the disk diffusion assay and MIC values [43]. The disk diffusion assay was performed in TSB and LB plates to examine the antibacterial activity for screening the marker compounds (**1**–**8**). The marker compounds were prepared in DMSO (1%) at 100 μg/mL (subinhibitory concentrations of 5, 10, 25, and 50 μg/mL). The assay was performed with LB and TSB plates. Sterile beads were used to inoculate the surface of each plate to ensure homogeneous bacterial growth. Sample pellet disks were placed on the surface of the agar plates at equal distances. The inhibition was measured using a ruler after 24 h incubation at 30 and 37 °C. MIC assay was performed in a 96-well plate to examine the antibacterial effects of three marker compounds (**1**, **3**, and **7**). Following serial dilution of the compounds (**1**, **3**, and **7**) in 150 μL of Mueller–Hinton broth, the culture was inoculated at an optical density (OD) of 0.002. After incubation at 30 and 37 °C for 24 h with shaking, bacterial growth was measured at OD_600_ using a spectrophotometer (BioTek Synergy HTX).

## 4. Conclusions

In the present study, indigenous Korean *Salix* species (*S. triandra*, *S. chaenomeloides*, *S. gracilistyla*, *S. koriyanagi*, *S. koreensis*, *S. pseudolasiogyne*, *S. caprea*, and *S. rorida*) were phytochemically investigated, followed by the isolation of their marker compounds: 3-(4-hydroxyphenyl)propyl β-D-glucopyranoside (**1**) from *S. triandra*, 2-*O*-acetylsalicin (**2**) from *S. chaenomeloides*, 1-*O*-*p*-coumaroyl glucoside (**3**) from *S. gracilistyla*, picein (**4**) from *S. koriyanagi*, isograndidentatin B (**5**) from *S. koreensis*, 2′-*O*-acetylsalicortin (**6**) from *S. pseudolasiogyne*, dihydromyricetin (**7**) from *S. caprea*, and salicin (**8**) from *S. rorida*. The chemical structures of these compounds were also determined via NMR spectroscopy and HR-ESI-MS analysis. Antimicrobial tests of the marker compounds (**1**–**8**) using plant pathogenic fungi and bacteria revealed that dihydromyricetin (**7**) exhibited antibacterial activity against *S. aureus*, inducing 32.4% inhibition at a final concentration of 125 μg/mL, with an MIC_50_ value of 250 μg/mL.

## Figures and Tables

**Figure 4:**
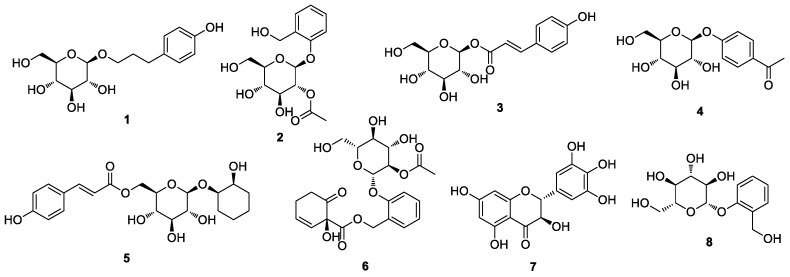
Chemical structures of the marker compounds **1–8**.

**Table 1:** Antibacterial activities of the selected marker compounds **1**, **3,** and **7** against *Staphylococcus aureus*.

Compound	Concentration (μg/mL)	Inhibition (%)	MIC_50_ (μg/mL)	MIC_90_ (μg/mL)
**1**	125	1.9		
**3**		1.3		
**7**		32.4	250	500
Gentamicin ^a^	7.8	96.5	1.95	3.91

^a^ Positive control.

## Supplementary Materials

The following supporting information can be downloaded at https://www.mdpi.com/article/10.3390/plants12010104/s1. Figure S1: The ^1^H NMR spectrum of Compound **1** (CD_3_OD, 850 MHz); Figure S2: The HR-ESIMS data for Compound **1**; Figure S3: The ^1^H NMR spectrum of Compound **2** (CD_3_OD, 850 MHz); Figure S4: The HR-ESIMS data for Compound **2**; Figure S5: The ^1^H NMR spectrum of Compound **3** (DMSO-*d_6_*, 850 MHz); Figure S6: The HR-ESIMS data for Compound **3**; Figure S7: The ^1^H NMR spectrum of Compound **4** (DMSO-*d_6_*, 850 MHz); Figure S8: The HR-ESIMS data for Compound **4**; Figure S9: The ^1^H NMR spectrum of Compound **5** (CD_3_OD, 850 MHz); Figure S10: The HR-ESIMS data for Compound **5**; Figure S11: The ^1^H NMR spectrum of Compound **6** (CD_3_OD, 850 MHz); Figure S12: The HR-ESIMS data for Compound **6**; Figure S13: The ^1^H NMR spectrum of Compound **7** (DMSO-*d_6_*, 850 MHz); Figure S14: The HR-ESIMS data for Compound **7**; Figure S15: The ^1^H NMR spectrum of Compound **8** (DMSO-*d_6_*, 850 MHz); Figure S16: The HR-ESIMS data for Compound **8**.

## References

[1] Mahdi J.G., Mahdi A.J., Mahdi A.J., Bowen I.D. (2006). The historical analysis of aspirin discovery, its relation to the willow tree and antiproliferative and anticancer potential. Cell Prolif..

[2] Du Q., Jerz G., Shen L., Xiu L., Winterhalter P. (2007). Isolation and structure determination of a lignan from the bark of *Salix alba*. Nat. Prod. Res..

[3] Freischmidt A., Jurgenliemk G., Kraus B., Okpanyi S.N., Muller J., Kelber O., Weiser D., Heilmann J. (2012). Contribution of flavonoids and catechol to the reduction of ICAM-1 expression in endothelial cells by a standardised Willow bark extract. Phytomedicine Int. J. Phytother. Phytopharm..

[4] Sultana S., Saleem M. (2004). *Salix caprea* inhibits skin carcinogenesis in murine skin: Inhibition of oxidative stress, ornithine decarboxylase activity and DNA synthesis. J. Ethnopharmacol..

[5] Li X., Liu Z., Zhang X.F., Wang L.J., Zheng Y.N., Yuan C.C., Sun G.Z. (2008). Isolation and characterization of phenolic compounds from the leaves of *Salix matsudana*. Molecules.

[6] Alam M.S., Kaur G., Jabbar Z., Javed K., Athar M. (2006). Evaluation of antioxidant activity of *Salix caprea* flowers. Phytother. Res. PTR.

[7] Han L.K., Sumiyoshi M., Zhang J., Liu M.X., Zhang X.F., Zheng Y.N., Okuda H., Kimura Y. (2003). Anti-obesity action of Salix matsudana leaves (Part 1). Anti-obesity action by polyphenols of *Salix matsudana* in high fat-diet treated rodent animals. Phytother. Res. PTR.

[8] Yang H., Lee S.H., Sung S.H., Kim J., Kim Y.C. (2013). Neuroprotective compounds from *Salix pseudo-lasiogyne* twigs and their anti-amnesic effects on scopolamine-induced memory deficit in mice. Planta Med..

[9] Gligorić E., Igić R., Suvajdžić L., Grujić-Letić N. (2019). Species of the genus Salix L.: Biochemical screening and molecular docking approach to potential acetylcholinesterase inhibitors. Appl. Sci..

[10] Kim C.S., Subedi L., Park K.J., Kim S.Y., Choi S.U., Kim K.H., Lee K.R. (2015). Salicin derivatives from *Salix glandulosa* and their biological activities. Fitoterapia.

[11] Kim C.S., Kwon O.W., Kim S.Y., Choi S.U., Kim J.Y., Han J.Y., Lee K.R. (2014). Phenolic glycosides from the twigs of *Salix glandulosa*. J. Nat. Prod..

[12] Jeong Y.U., Park Y.J. (2019). Effect of *Ganoderma lucidum* Solid-state Fermented *Salix gracilistyla* Extract on Type I Procollagen Biosynthesis in HDFn Cells. Korean J. Mycol..

[13] Ryu J.H., Ahn H., Kim J.Y., Kim Y.K. (2003). Inhibitory activity of plant extracts on nitric oxide synthesis in LPS-activated macrophages. Phytother. Res..

[14] Jeong Y.-U., Park Y.-J. (2018). Studies on antioxidant and whitening activities of *Salix gracilistyla* extracts. J. Soc. Cosmet. Sci. Korea.

[15] Jung B.N., Park J.H., Shin H.D. (2020). First report of *Rhytisma filamentosum* causing tar-spot disease on *Salix koriyanagi*. For. Pathol..

[16] Yoon A., Oh H.E., Kim S.Y., Park Y.G. (2021). Plant growth regulators and rooting substrates affect growth and development of Salix koriyanagi cuttings. Rhizosphere.

[17] Ahn Y., Yang Y., Chun S. (2001). A study on the distribution patterns of Salicaceae species at the An-sung stream-refered to Woldongcheon, Yokjungcheon, Joyoungcheon and Gisolcheon. Kor. J. Env. Eco.

[18] Park Y. (2006). A study about control of gingivitis and plaque of dentifrice containing tranexamic acid and willow tree bark. J Korean Acad. Dent. Health.

[19] Hostanska K., Jürgenliemk G., Abel G., Nahrstedt A., Saller R. (2007). Willow bark extract (BNO1455) and its fractions suppress growth and induce apoptosis in human colon and lung cancer cells. Cancer Detect. Prev..

[20] Kim E.-J., Kim M.H. (2016). Anti-oxidant and anti-inflammatory effects of Salix koreensis Andersson in DC. leaf methanol extract in vitro models. CELLMED.

[21] Kim M.H. (2018). Antioxidant activity and anti-inflammatory effects of Salix Koreensis Andersson branches extracts. J. Korean Soc. Food Cult..

[22] Kim S., Min J., Kang H. (2020). Hepatoprotective effects of willow (*Salix koreensis* ANDERSS.) branch extracts against cytotoxicity induced by tert-butyl hydroperoxide in human hepatoma cells. J. Korean Soc. Food Sci. Nutr..

[23] Ko K.S., Jeon E.S. (2003). Fern-Allies and Seed-Bearing Plants of Korea.

[24] Lee M., Lee S.H., Kang J., Yang H., Jeong E.J., Kim H.P., Sung S.H. (2013). Salicortin-derivatives from Salix pseudo-lasiogyne twigs inhibit adipogenesis in 3T3-L1 cells via modulation of C/EBPα and SREBP1c dependent pathway. Molecules.

[25] Noleto-Dias C., Ward J.L., Bellisai A., Lomax C., Beale M.H. (2018). Salicin-7-sulfate: A new salicinoid from willow and implications for herbal medicine. Fitoterapia.

[26] Lee S.R., Kang H., Yoo M.J., Yu J.S., Lee S., Yi S.A., Beemelmanns C., Lee J., Kim K.H. (2020). Anti-adipogenic pregnane steroid from a Hydractinia-associated fungus, *Cladosporium sphaerospermum* SW67. Nat. Prod. Sci..

[27] Yu J.S., Park M., Pang C., Rashan L., Jung W.H., Kim K.H. (2020). Antifungal phenols from *Woodfordia uniflora* collected in Oman. J. Nat. Prod..

[28] Lee S., Kim C.S., Yu J.S., Kang H., Yoo M.J., Youn U.J., Ryoo R., Bae H.Y., Kim K.H. (2021). Ergopyrone, a Styrylpyrone-Fused Steroid with a Hexacyclic 6/5/6/6/6/5 Skeleton from a Mushroom *Gymnopilus orientispectabilis*. Org. Lett..

[29] Lee K.H., Kim J.K., Yu J.S., Jeong S.Y., Choi J.H., Kim J.C., Ko Y.J., Kim S.H., Kim K.H. (2021). Ginkwanghols A and B, osteogenic coumaric acid-aliphatic alcohol hybrids from the leaves of Ginkgo biloba. Arch. Pharmacal Res..

[30] Yu J.S., Li C., Kwon M., Oh T., Lee T.H., Kim D.H., Ahn J.S., Ko S.-K., Kim C.S., Cao S. (2020). Herqueilenone A, a unique rearranged benzoquinone-chromanone from the hawaiian volcanic soil-associated fungal strain *Penicillium herquei* FT729. Bioorg. Chem..

[31] Ha J.W., Kim J., Kim H., Jang W., Kim K.H. (2020). Mushrooms: An important source of natural bioactive compounds. Nat. Prod. Sci..

[32] Lee S., Ryoo R., Choi J.H., Kim J.-H., Kim S.-H., Kim K.H. (2020). Trichothecene and tremulane sesquiterpenes from a hallucinogenic mushroom *Gymnopilus junonius* and their cytotoxicity. Arch. Pharm. Res..

[33] Akita H., Kawahara E., Kishida M., Kato K. (2006). Synthesis of naturally occurring β-D-glucopyranoside based on enzymatic β-glycosidation. J. Mol. Catal. B Enzym..

[34] Tawfeek N., Sobeh M., Hamdan D.I., Farrag N., Roxo M., El-Shazly A.M., Wink M. (2019). Phenolic compounds from *Populus alba* L. and *Salix subserrata* Willd. (Salicaceae) counteract oxidative stress in *Caenorhabditis elegans*. Molecules.

[35] Whang W.K., Chang Y.S., Kim I.H. (1995). Phenolic compounds from the bark of *Salix Gilgiana*. Yakhak Hoeji.

[36] Jeon S.H., Chun W., Choi Y.J., Kwon Y.S. (2008). Cytotoxic constituents from the bark of *Salix hulteni*. Arch. Pharmacal Res..

[37] Si C.L., Kim J.K., Bae Y.S., Li S.M. (2009). Phenolic compounds in the leaves of *Populus ussuriensis* and their antioxidant activities. Planta Med..

[38] Reichardt P.B., Merken H.M., Clausen T.P., Wu J. (1992). Phenolic glycosides from Salix lasiandra. Journal of Natural Products. J. Nat. Prod..

[39] Du Q., Cai W., Xia M., Ito Y. (2002). Purification of (+)-dihydromyricetin from leaves extract of *Ampelopsis grossedentata* using high-speed countercurrent chromatograph with scale-up triple columns. J. Chromatogr. A.

[40] Lindroth R.L., Hsia M.S., Scriber J.M. (1987). Characterization of phenolic glycosides from quaking aspen. Biochem. Syst. Ecol..

[41] Choi G.J., Jang K.S., Choi Y.H., Yu J.H., Kim J.C. (2010). Antifungal activity of lower alkyl fatty acid esters against powdery mildews. Plant Pathol. J..

[42] Hong J.H., Lee J., Min M., Ryu S.M., Lee D., Kim G.H., Kim J.J. (2014). 6-Pentyl-α-pyrone as an anti-sapstain compound produced by *Trichoderma gamsii* KUC1747 inhibits the germination of ophiostomatoid fungi. Holzforschung.

[43] Yu J.S., Kim J.H., Rashan L., Kim I., Lee W., Kim K.H. (2021). Potential Antimicrobial Activity of Galloyl-Flavonoid glycosides From *Woodfordia uniflora* Against Methicillin-Resistant *Staphylococcus aureus*. Front. Microbiol..

