# Supplementary material for: Phytochemical Investigation of Marker Compounds from Indigenous Korean Salix Species and Their Antimicrobial Effects
Source: Plants (Basel). 2022 Dec 26;12(1):104. doi: 10.3390/plants12010104 (PMC9824127; doi:10.3390/plants12010104)

Supplementary data

Phytochemical Investigation of Marker Compounds from Indigenous Korean *Salix* Species and Their Antimicrobial Effects

Yoon Seo Jang [1,†], Da Eun Lee [1,†], Joo-Hyun Hong [1], Kyung Ah Kim [1], Bora Kim [1], Yeo Rang Cho [1], Moon-Jin Ra [2], Sang-Mi Jung [2], Jeong-Nam Yu [3], Seongpil An [4,5,*] and Ki Hyun Kim [1,*]

1 School of Pharmacy, Sungkyunkwan University, Suwon 16419, Republic of Korea
2 Hongcheon Institute of Medicinal Herb, Hongcheon-gun, Gangwon-do 25142, Republic of Korea
3 Nakdonggang National Institute of Biological Resources, Sangju, Gyeongsangbuk-do 37242, Republic of Korea
4 SKKU Advanced Institute of Nanotechnology (SAINT), Sungkyunkwan University (SKKU), Suwon 16419, Republic of Korea
5 Department of Nano Engineering, Sungkyunkwan University (SKKU), Suwon 16419, Republic of Korea
* Correspondence: esan@skku.edu (S.A.); khkim83@skku.edu (K.H.K.); +82-31-290-7700 (K.H.K.)
† These authors contributed equally to this study.

Supporting Information Contents:

General experimental procedure……………………………………………………………………………………S3
Figure S1. The ^{1}H NMR spectrum of Compound **1** (CD_3OD, 850 MHz)……………………………………S4
Figure S2. The HR-ESIMS data for compound **1**……………………………………………………………S5
Figure S3. The ^{1}H NMR spectrum of Compound **2** (CD_3OD, 850 MHz)……………………………………S6
Figure S4. The HR-ESIMS data for Compound **2**……………………………………………………………S7
Figure S5. The ^{1}H NMR spectrum of Compound **3** (DMSO-d_6, 850 MHz)…………………………………S8
Figure S6. The HR-ESIMS data for Compound **3**……………………………………………………………S9
Figure S7. The ^{1}H NMR spectrum of Compound **4** (DMSO-d_6, 850 MHz)…………………………………S10
Figure S8. The HR-ESIMS data for Compound **4**……………………………………………………………S11
Figure S9. The ^{1}H NMR spectrum of Compound **5** (CD_3OD, 850 MHz)……………………………………S12
Figure S10. The HR-ESIMS data for Compound **5**……………………………………………………………S13
Figure S11. The ^{1}H NMR spectrum of Compound **6** (CD_3OD, 850 MHz)…………………………………S14
Figure S12. The HR-ESIMS data for Compound **6**……………………………………………………………S15
Figure S13. The ^{1}H NMR spectrum of Compound **7** (DMSO-d_6, 850 MHz)………………………………S16
Figure S14. The HR-ESIMS data for Compound **7**……………………………………………………………S17
Figure S15. The ^{1}H NMR spectrum of Compound **8** (DMSO-d_6, 850 MHz)………………………………S18
Figure S16. The HR-ESIMS data for Compound **8**……………………………………………………………S19

General experimental procedure

Optical rotations were measured using a JASCO P-2000 polarimeter (JASCO, Easton, MD, USA). Ultraviolet (UV) spectra were acquired on an Agilent 8453 UV-visible spectrophotometer (Agilent Technologies, Santa Clara, CA, USA). Nuclear magnetic resonance (NMR) spectra were recorded with a Bruker AVANCE III HD 850 NMR spectrometer with a 5 mm TCI CryoProbe operating at 850 MHz (^{1}H) and 212.5 MHz (^{13}C), with chemical shifts given in ppm (δ) for NMR analyses. Preparative high-performance liquid chromatography (HPLC) was performed using a Waters 1525 Binary HPLC pump with a Waters 996 Photodiode Array Detector (Waters Corporation, Milford, MA, USA) and an Agilent Eclipse C18 column (250 × 21.2 mm, 5 µm; flow rate: 5 mL/min; Agilent Technologies). Semi-preparative HPLC was performed using a Shimadzu Prominence HPLC System with SPD-20A/20AV Series Prominence HPLC UV-Vis detectors (Shimadzu, Tokyo, Japan) and a Phenomenex Luna C18 column (250 × 10 mm, 5 µm; flow rate: 2 mL/min; Phenomenex, Torrance, CA, USA). LC/MS analysis was performed on an Agilent 1200 Series HPLC system equipped with a diode array detector and 6130 Series ESI mass spectrometer using an analytical Kinetex C18 100 Å column (100 × 2.1 mm, 5 µm; flow rate: 0.3 mL/min; Phenomenex). The HR-ESIMS data were analyzed on an Agilent 1290 Infinity II UPLC coupled to a G6545B Q-TOF MS system with a dual ESI source (Agilent Technologies, USA) using an Agilent ZORBAX RRHD Eclipse Plus C_{18} column (50 × 2.1 mm, 1.8 µm, flow rate: 0.3 mL/min). Silica gel 60 (230-400 mesh; Merck, Darmstadt, Germany) and RP-C18 silica gel (Merck, 230-400 mesh) were used for column chromatography. The packing material for molecular sieve column chromatography was Sephadex LH-20 (Pharmacia, Uppsala, Sweden). Thin-layer chromatography (TLC) was performed with precoated silica gel F254 plates and RP-C18 F254s plates (Merck) and spots were detected under UV light or by heating after spraying with anisaldehyde-sulfuric acid.

Figure S1. The ^{1}H NMR spectrum of Compound **1** (CD_3OD, 850 MHz)

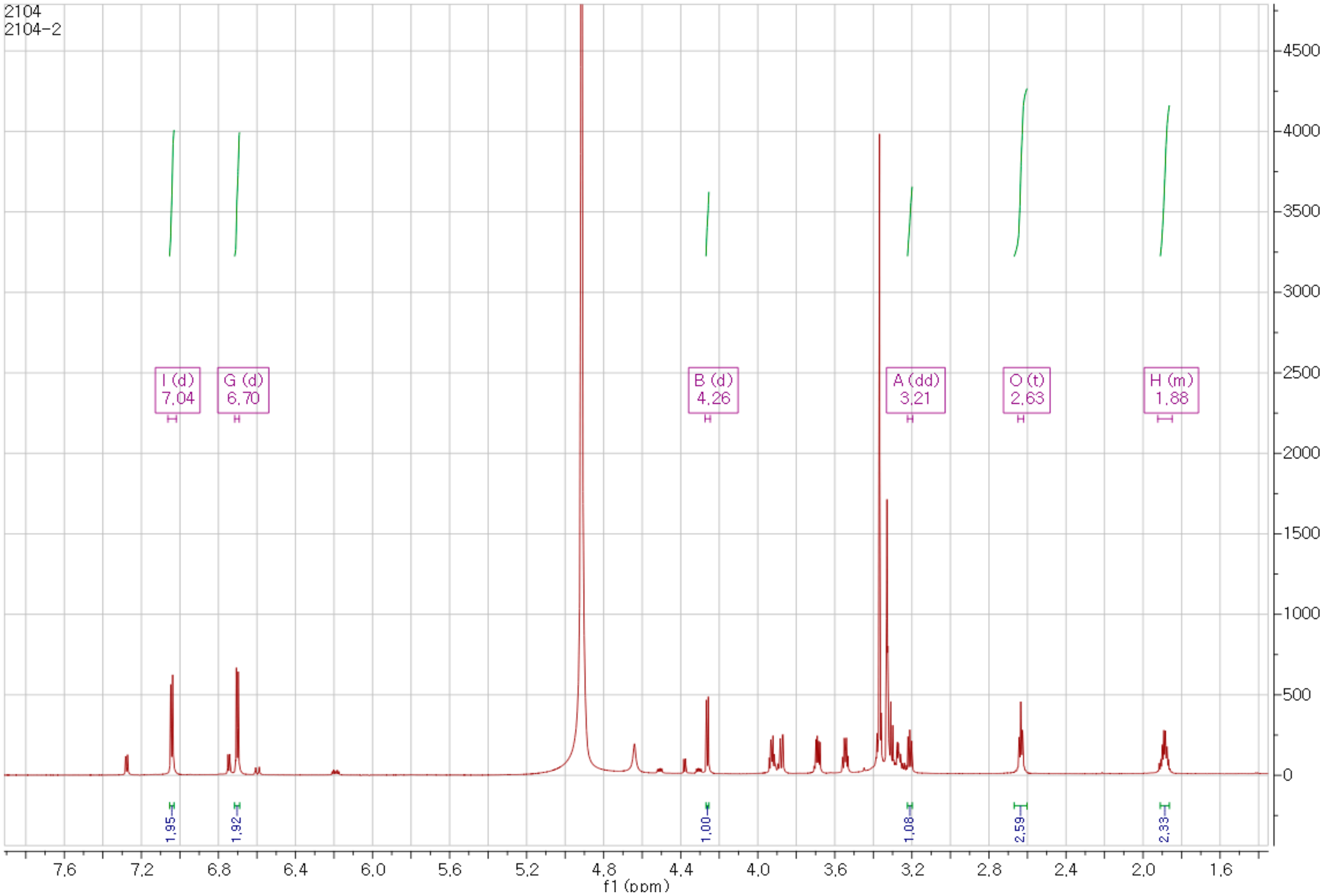

Figure S2. The HR-ESIMS data for Compound **1**

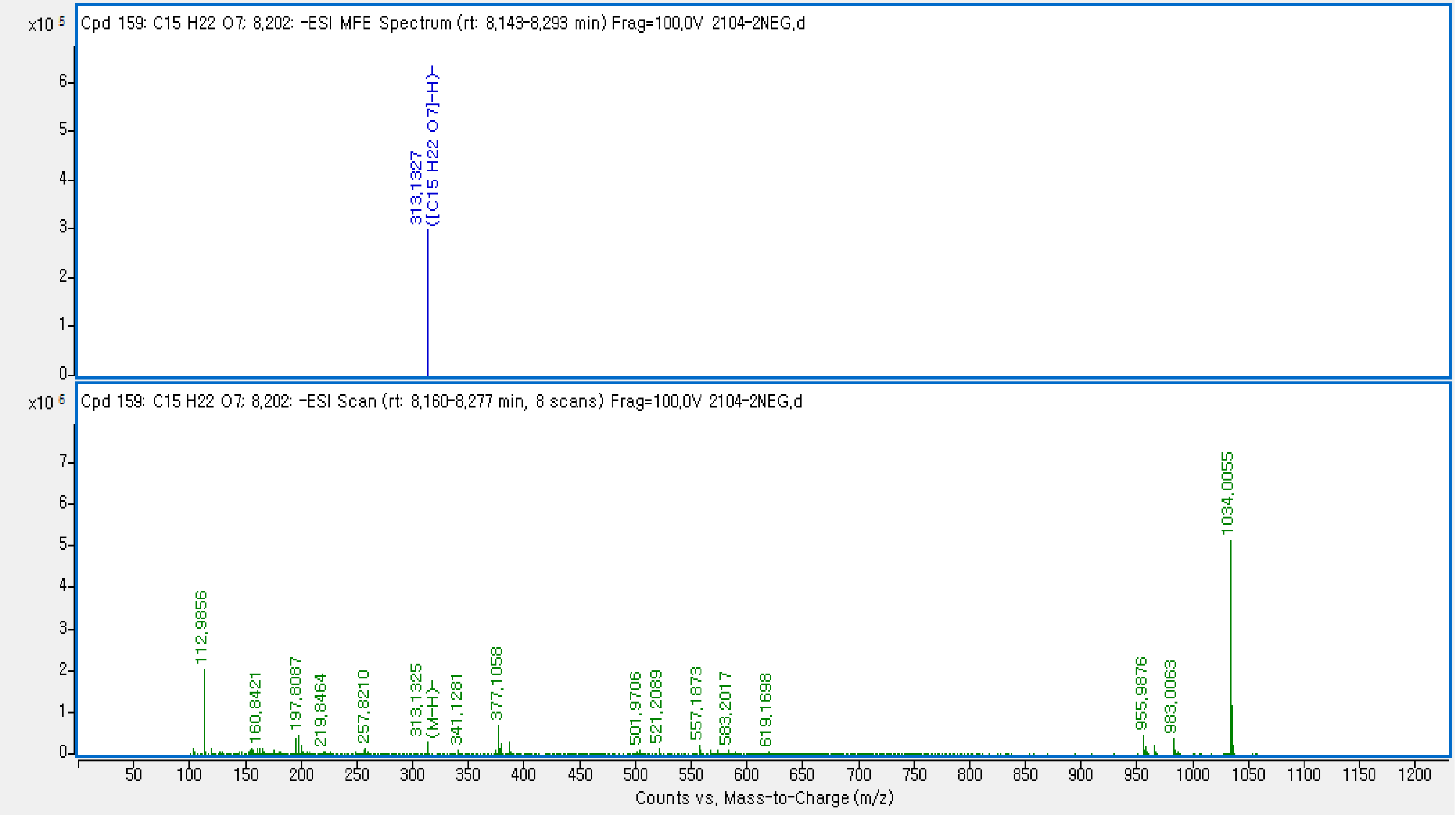

Figure S3. The ^{1}H NMR spectrum of Compound **2** (CD_3OD, 850 MHz)

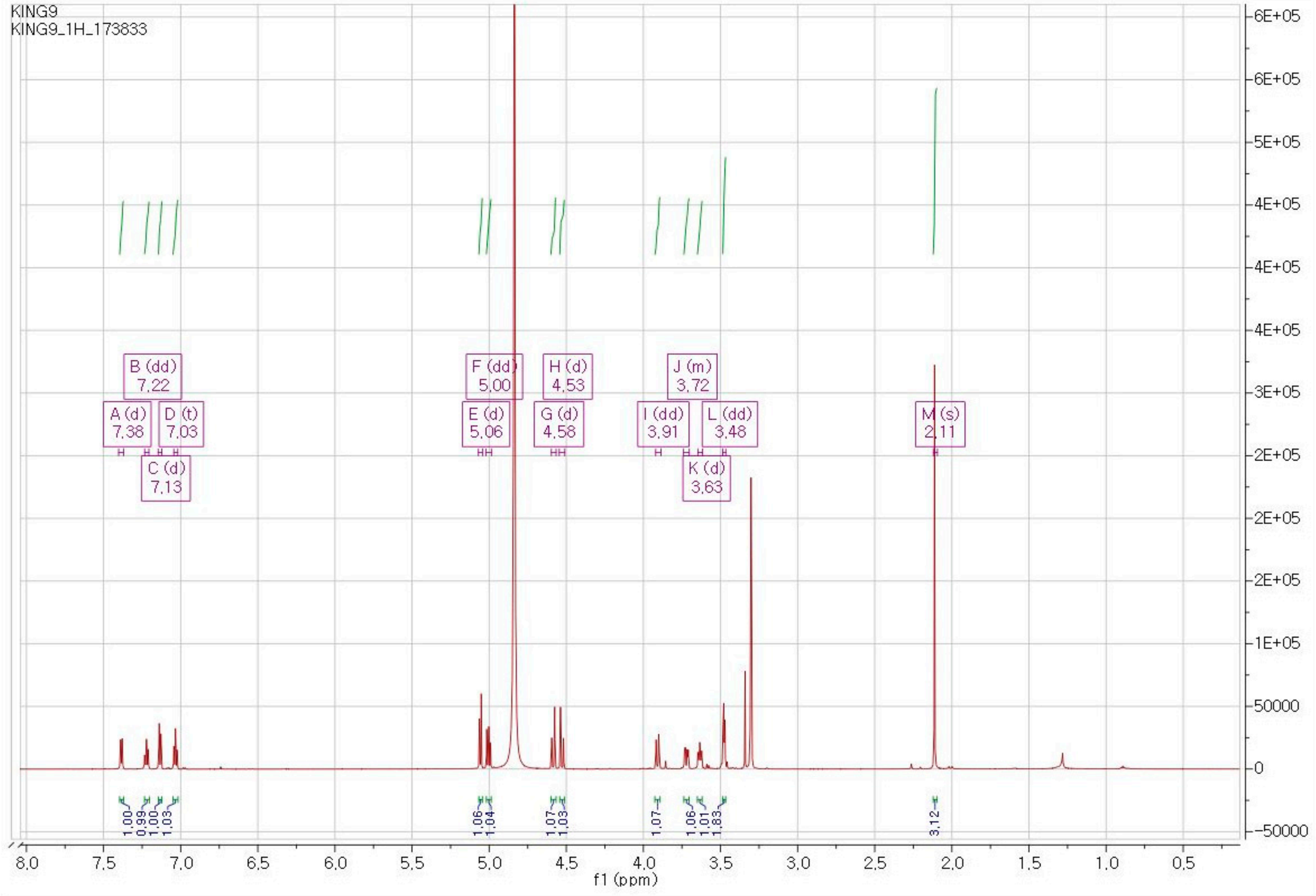

Figure S4. The HR-ESIMS data for compound **2**

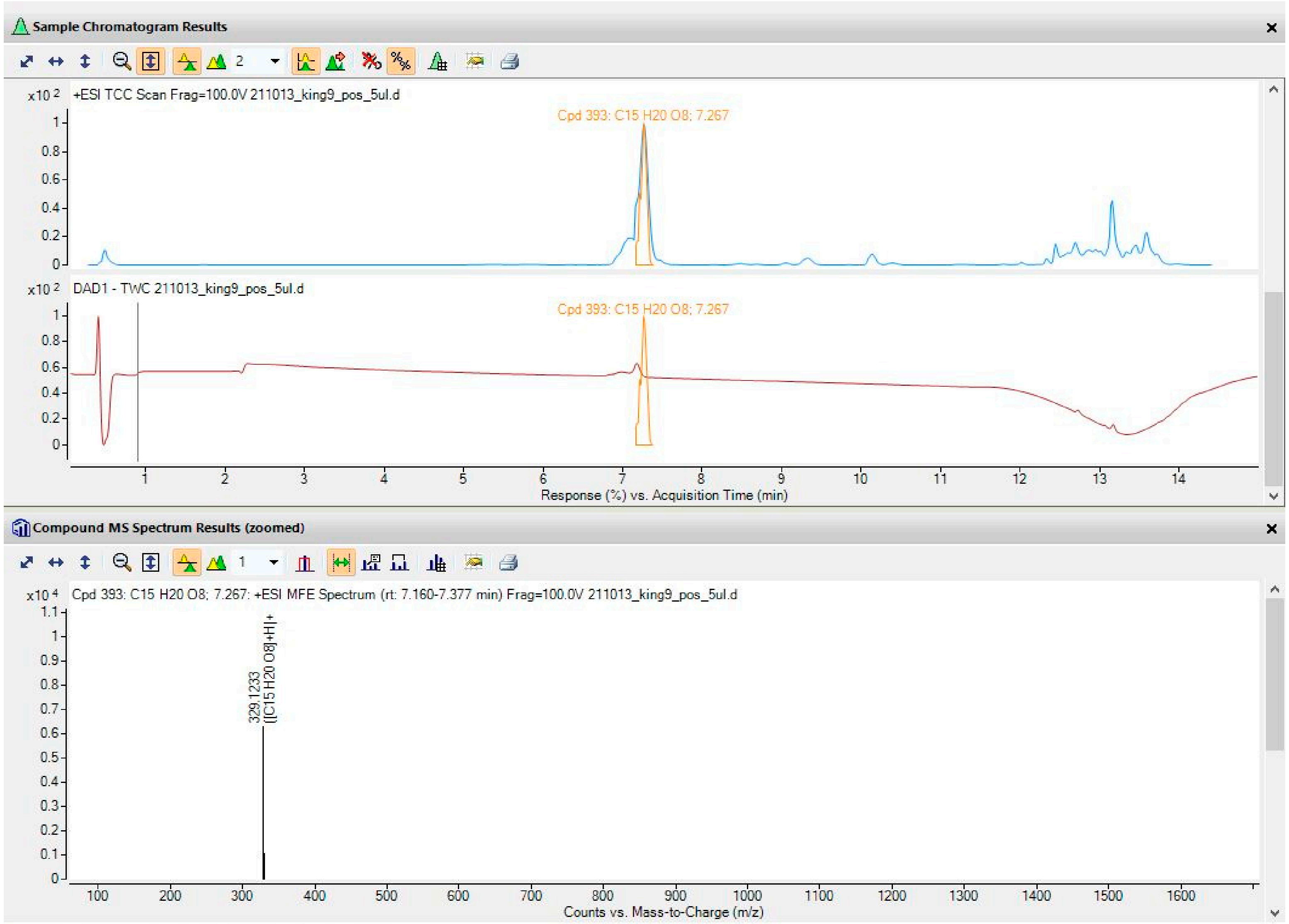

Figure S5. The ^{1}H NMR spectrum of Compound **3** (DMSO-d_6, 850 MHz)

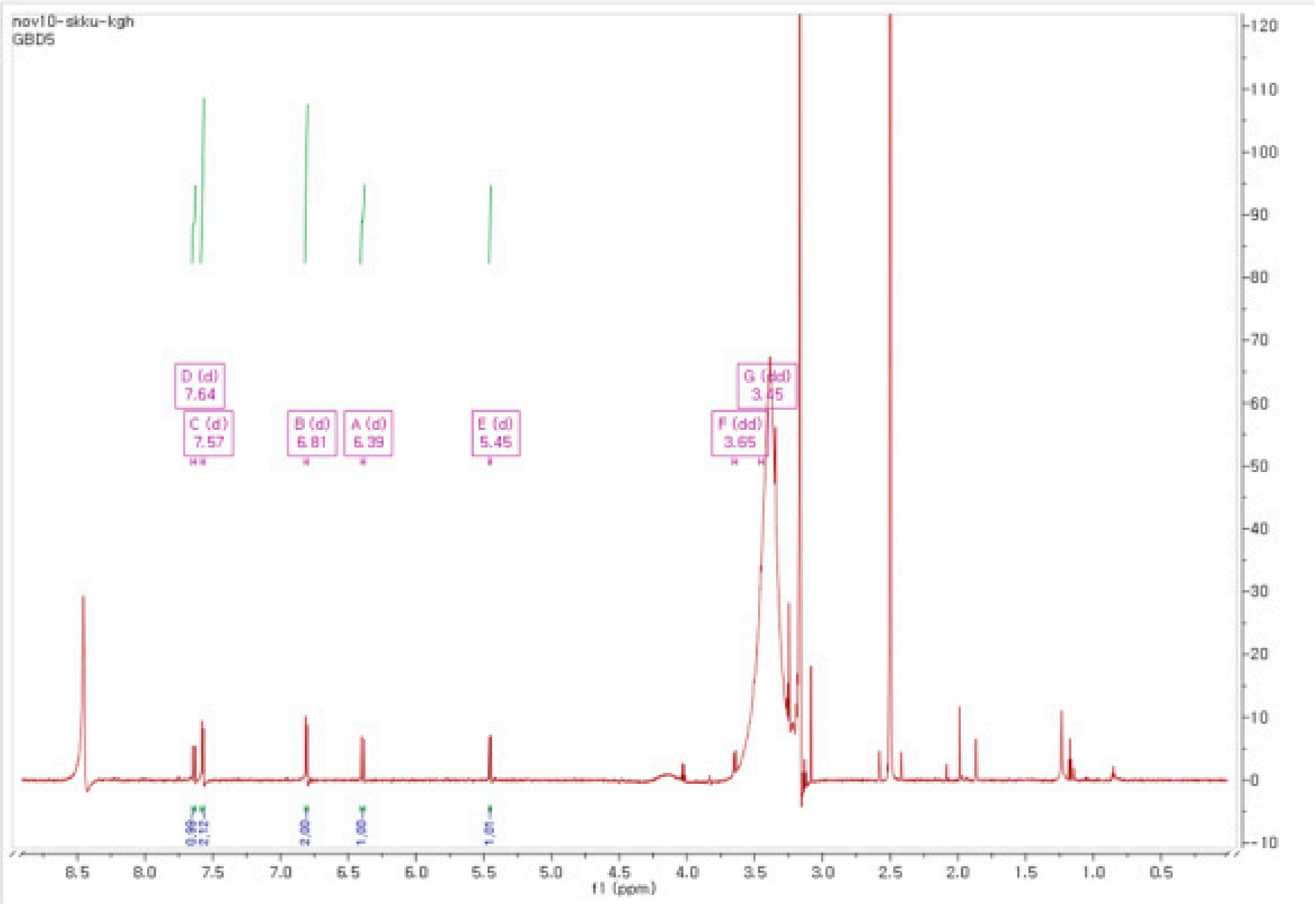

Figure S6. The HR-ESIMS data for Compound **3**

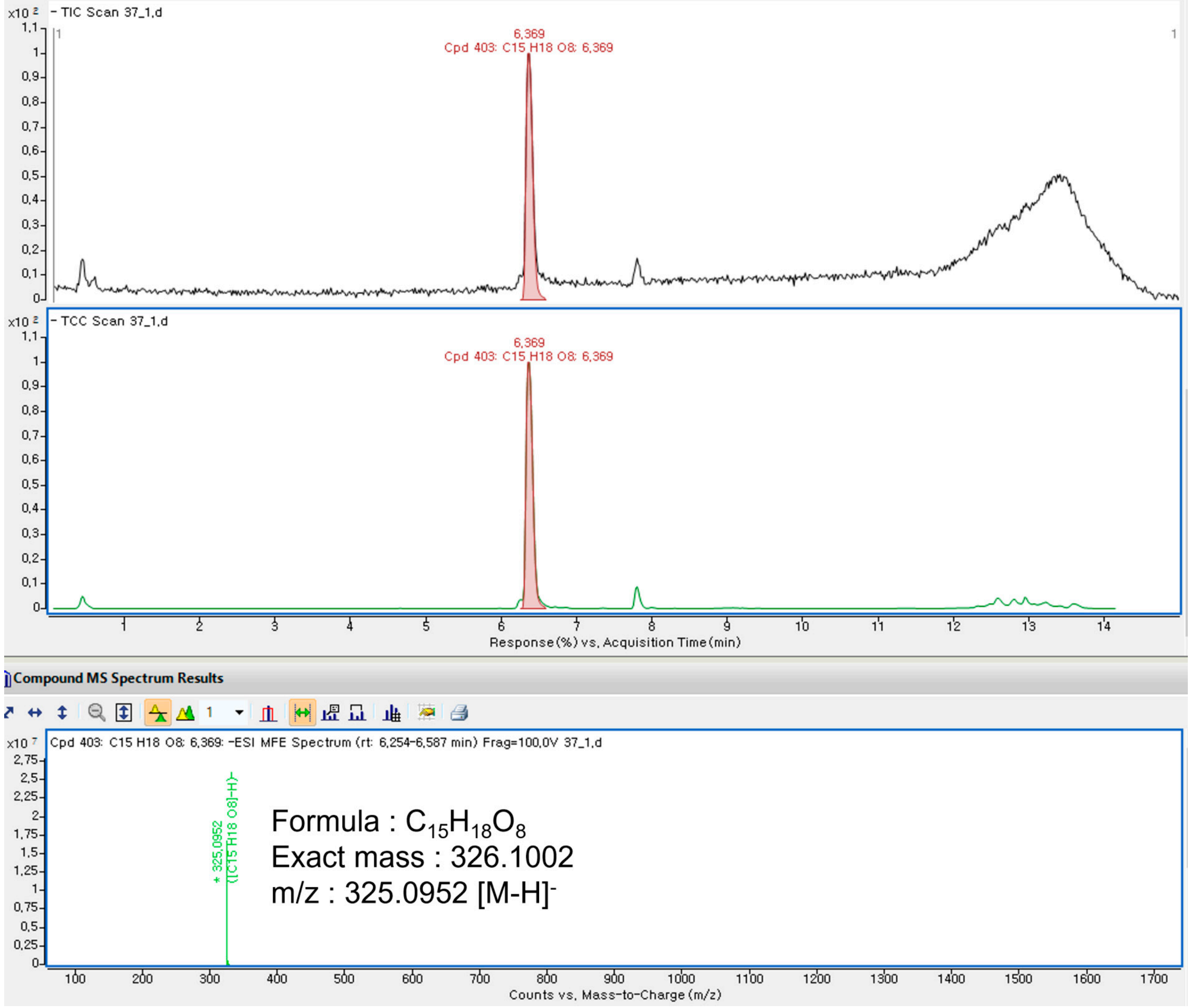

Figure S7. The ^{1}H NMR spectrum of Compound **4** (DMSO-d_6, 850 MHz)

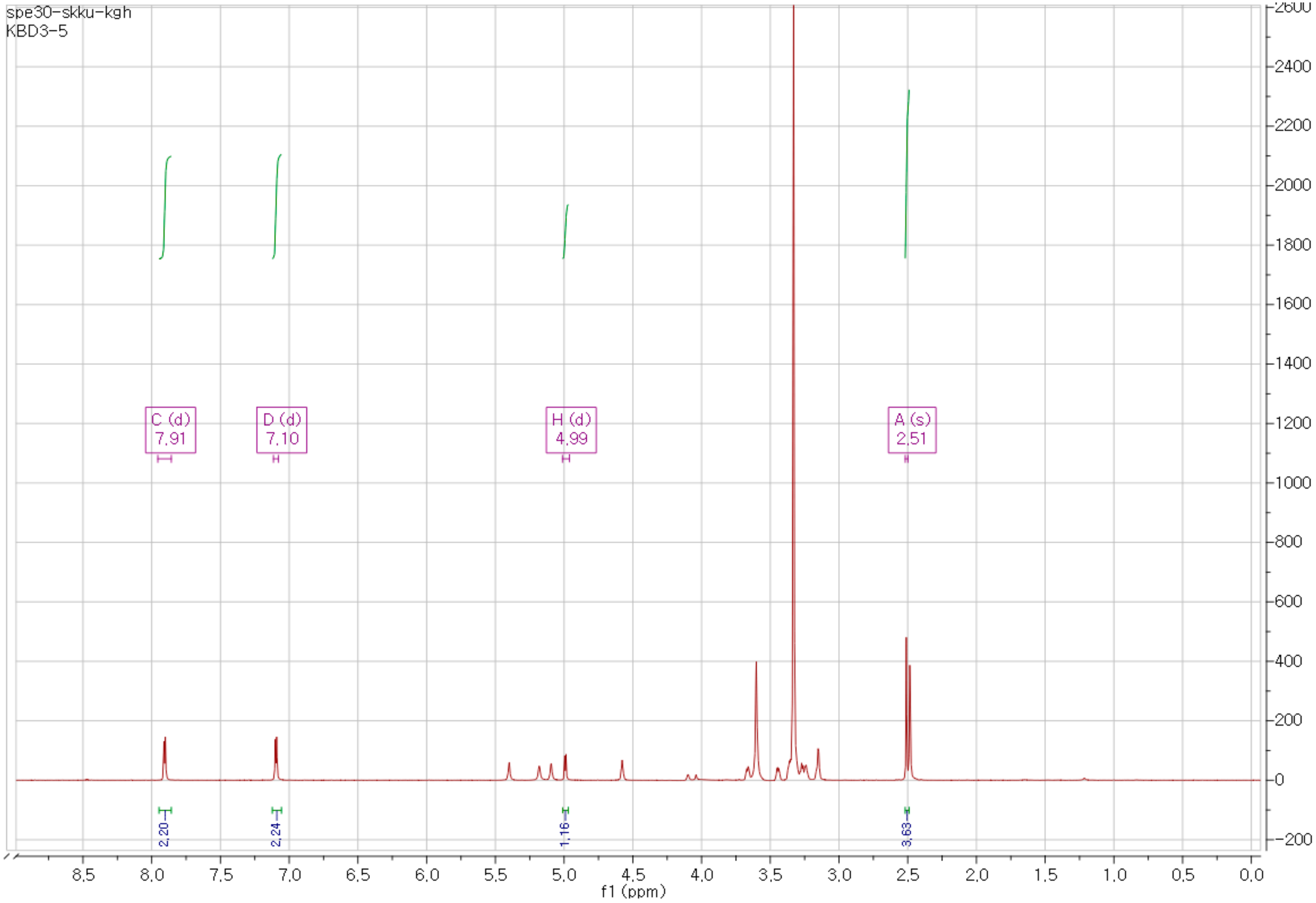

Figure S8. The HR-ESIMS data for Compound **4**

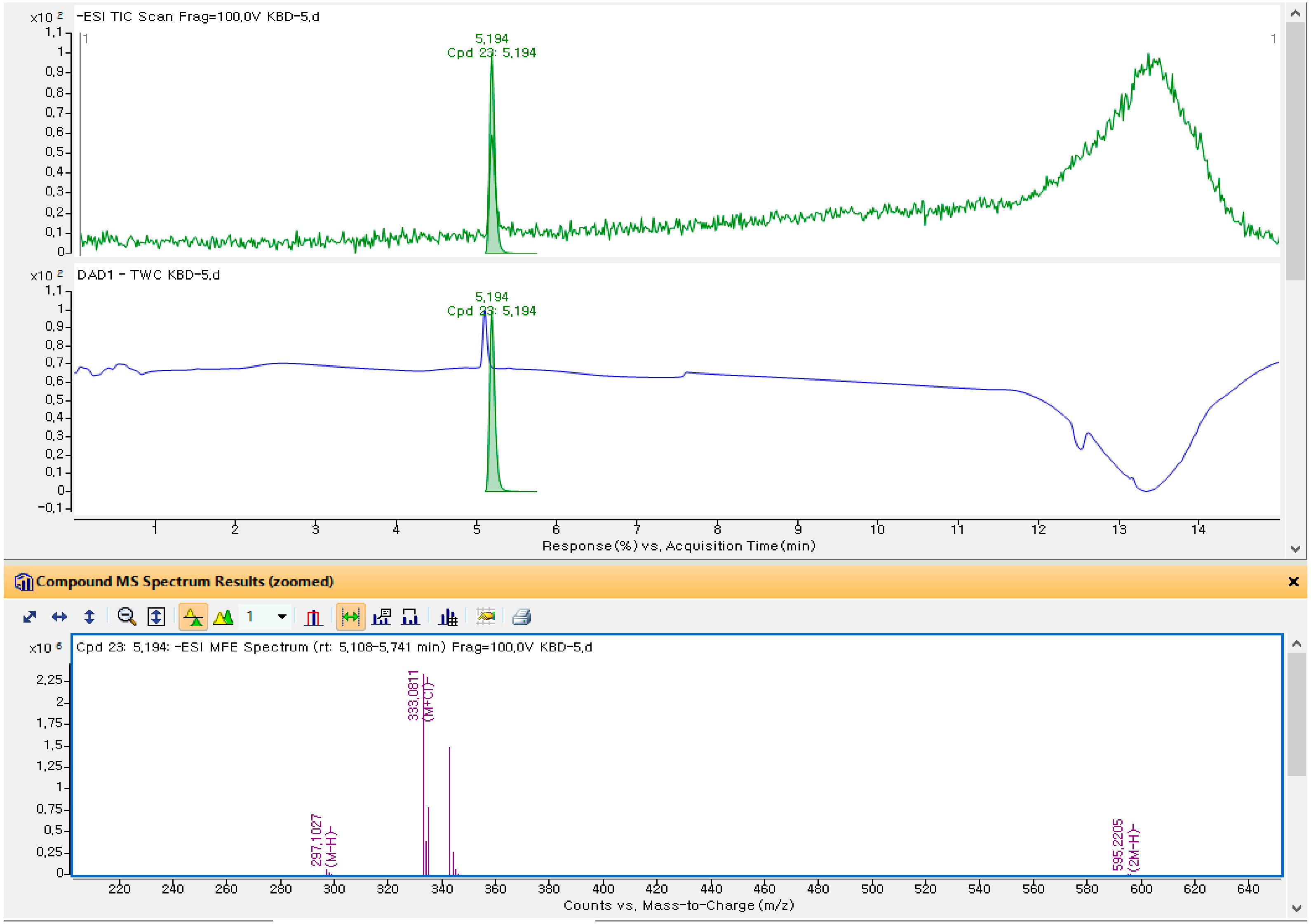

Figure S9. The ^{1}H NMR spectrum of Compound **5** (DMSO-d_6, 850 MHz)

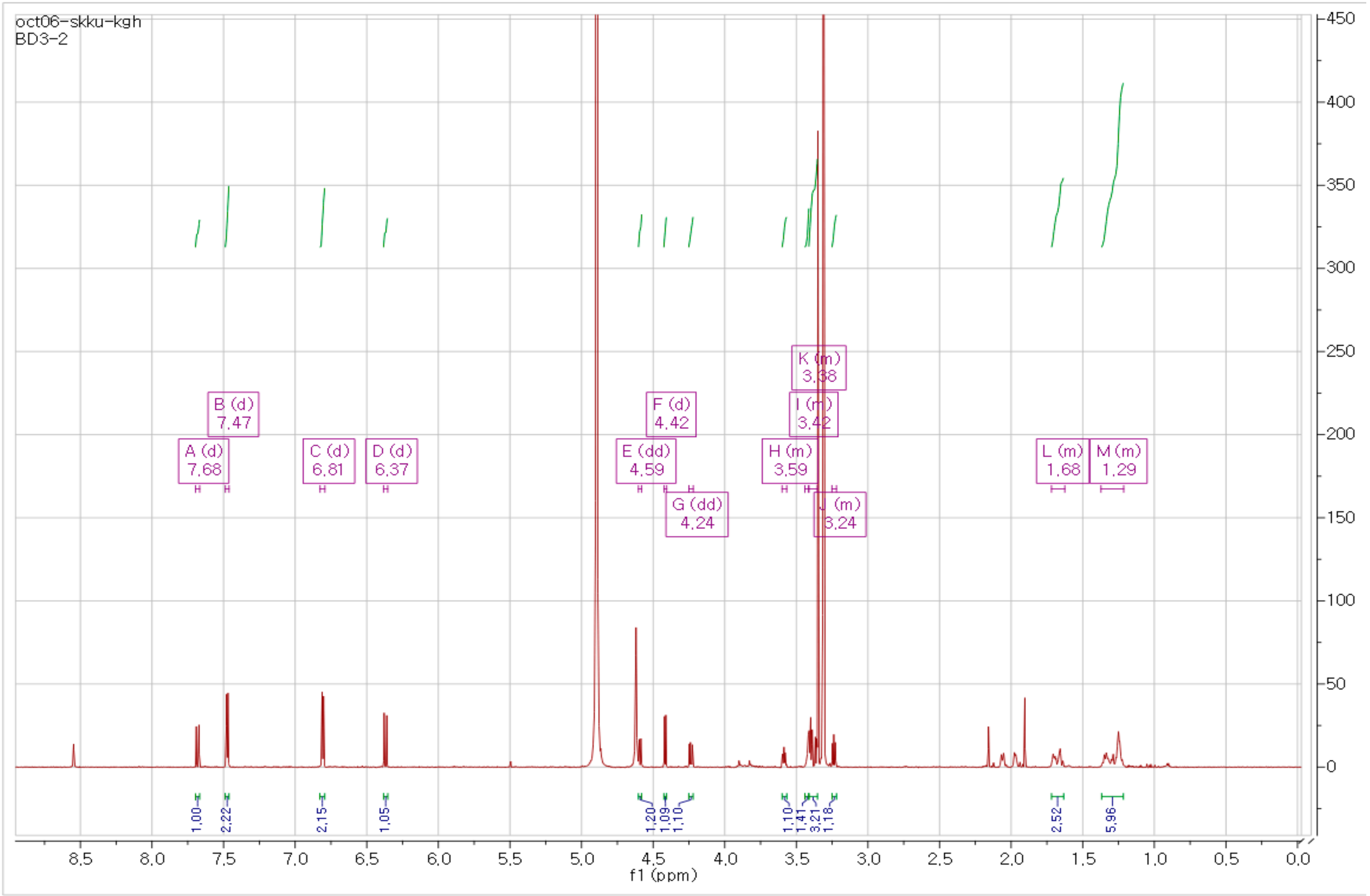

Figure S10. The HR-ESIMS data for Compound **5**

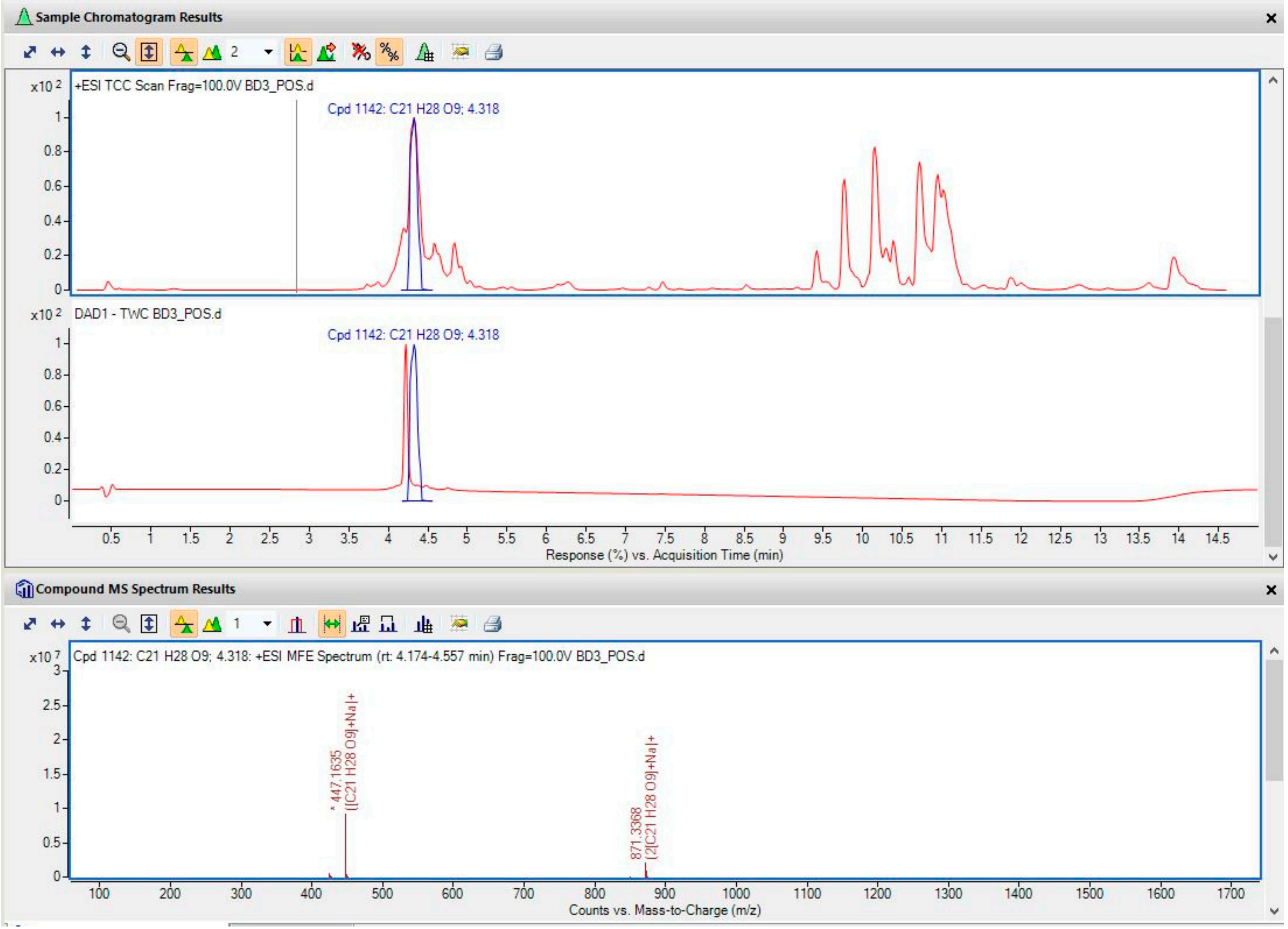

Figure S11. The ^{1}H NMR spectrum of Compound **6** (CD_3OD, 850 MHz)

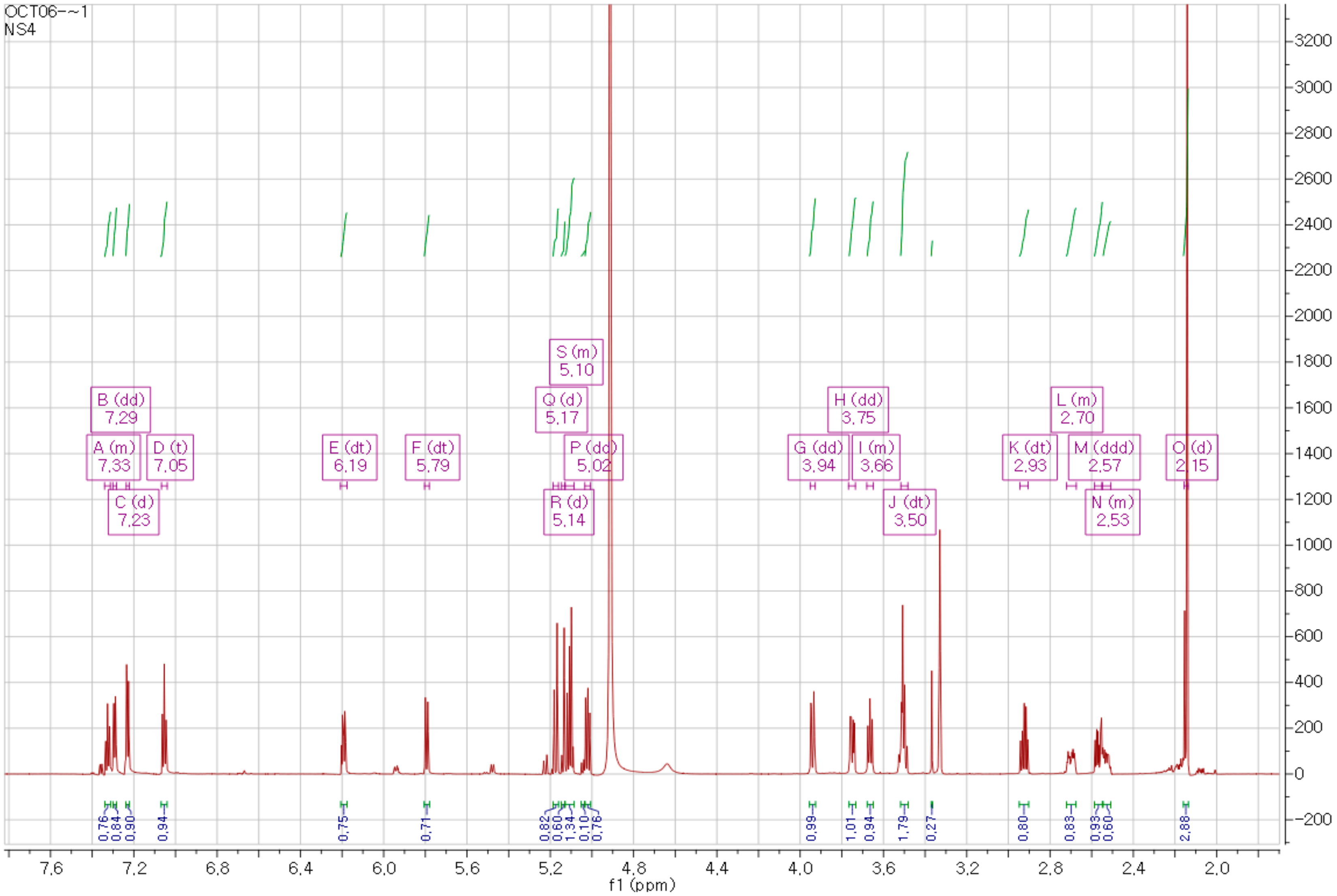

Figure S12. The HR-ESIMS data for Compound **6**

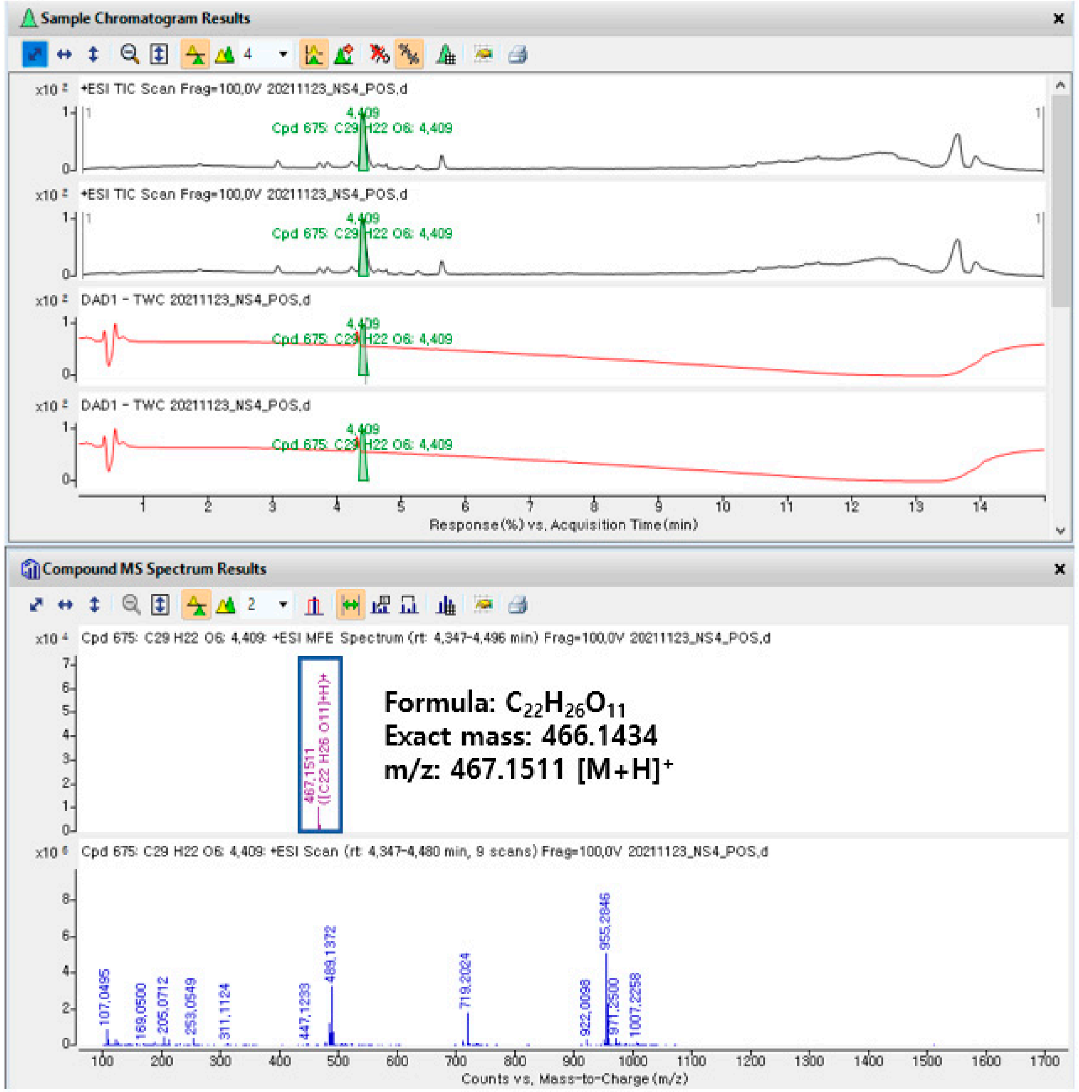

Figure S13. The ^{1}H NMR spectrum of Compound **7** (DMSO-d_6, 850 MHz)

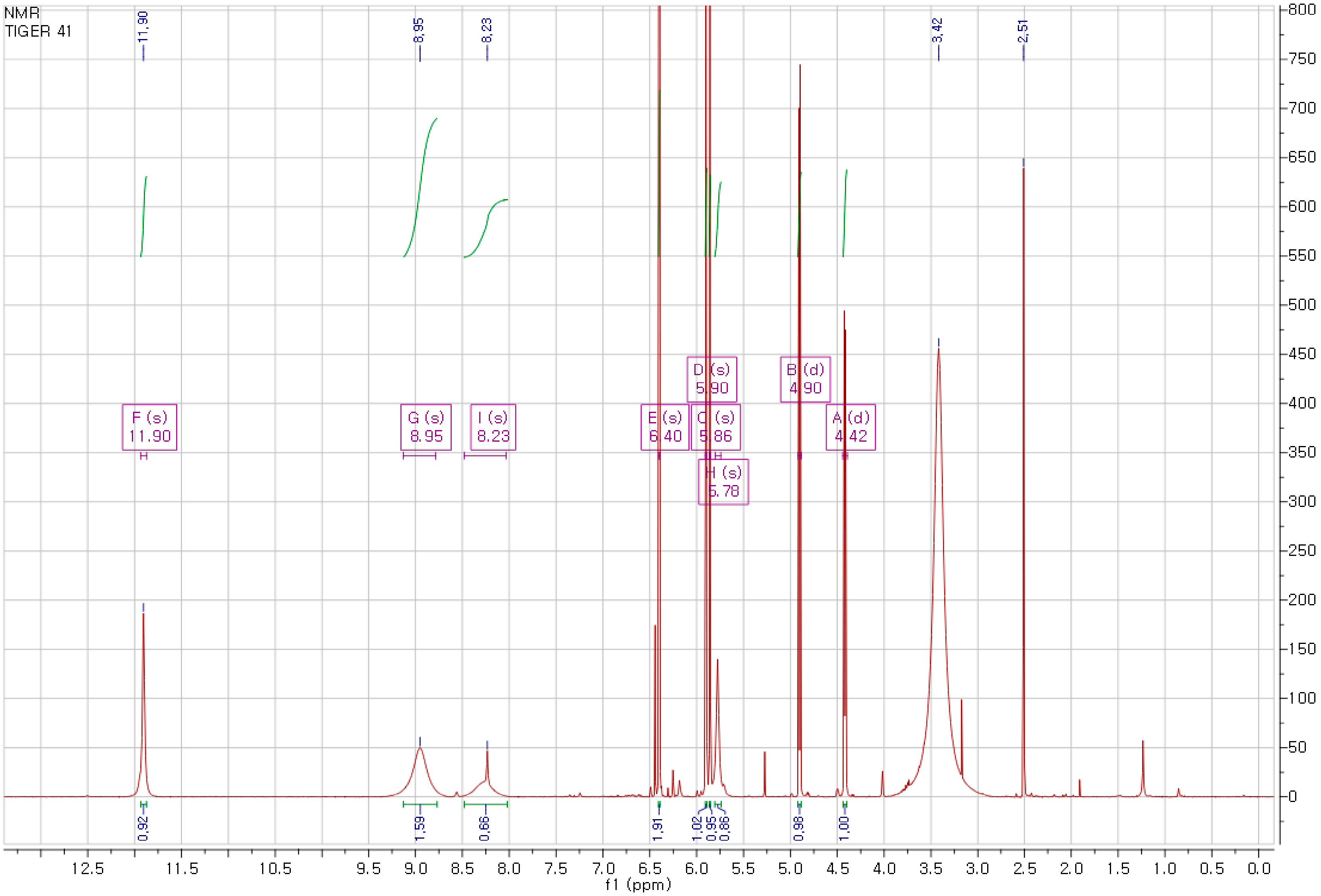

Figure S14. The HR-ESIMS data for Compound **7**

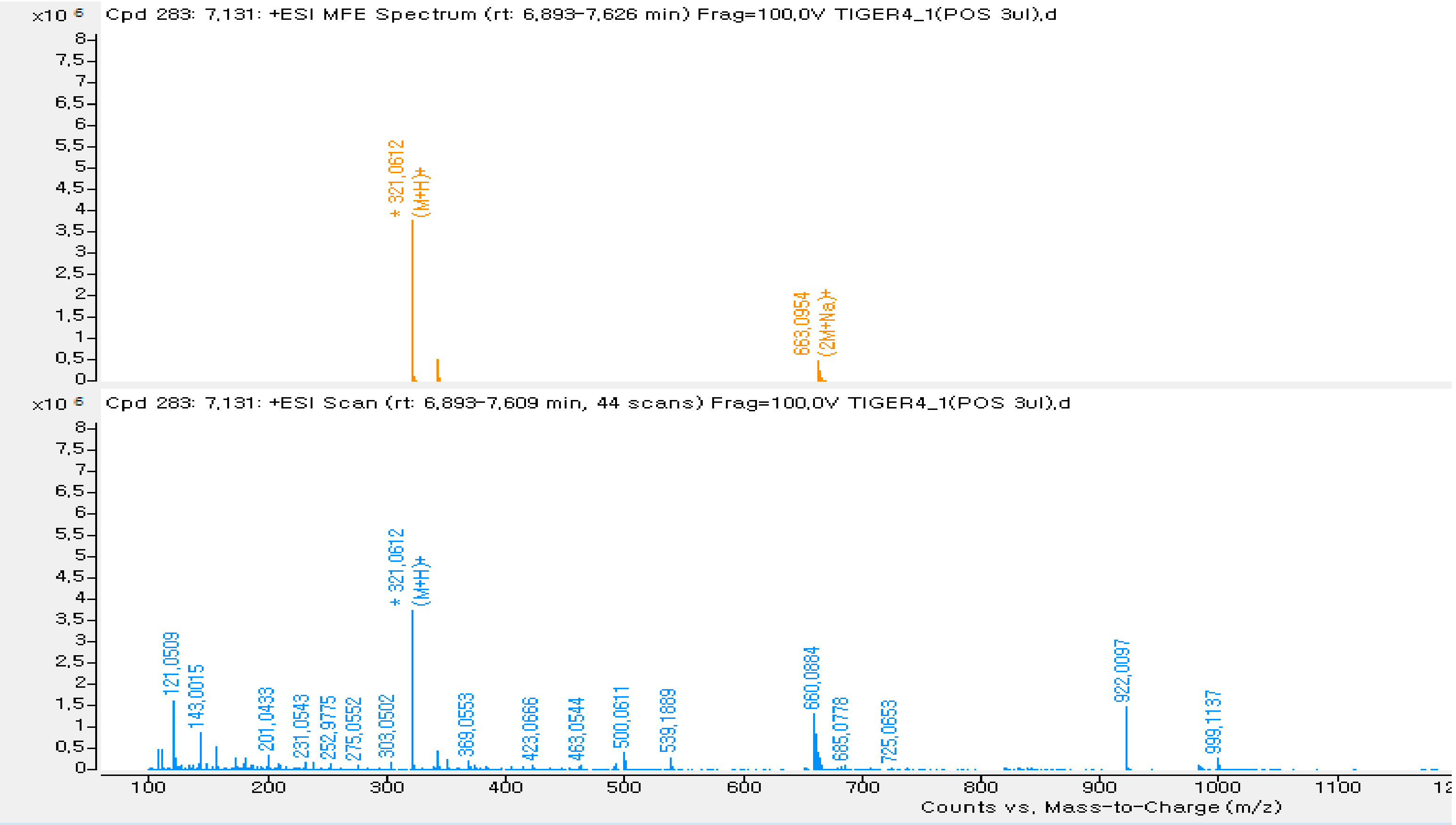

Figure S15. The ^{1}H NMR spectrum of Compound **8** (DMSO-d_6, 850 MHz)

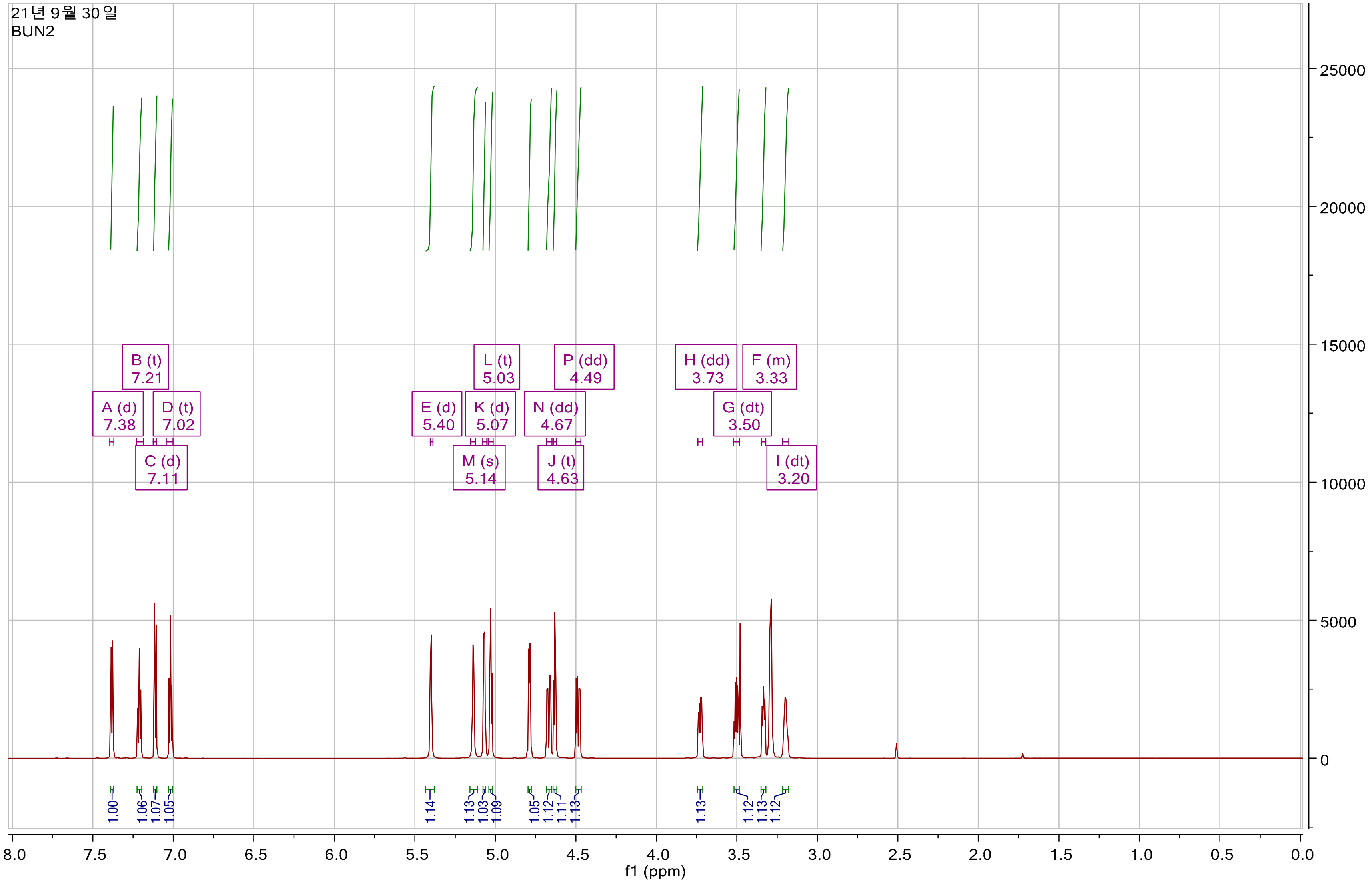

Figure S16. The HR-ESIMS data for Compound **8**

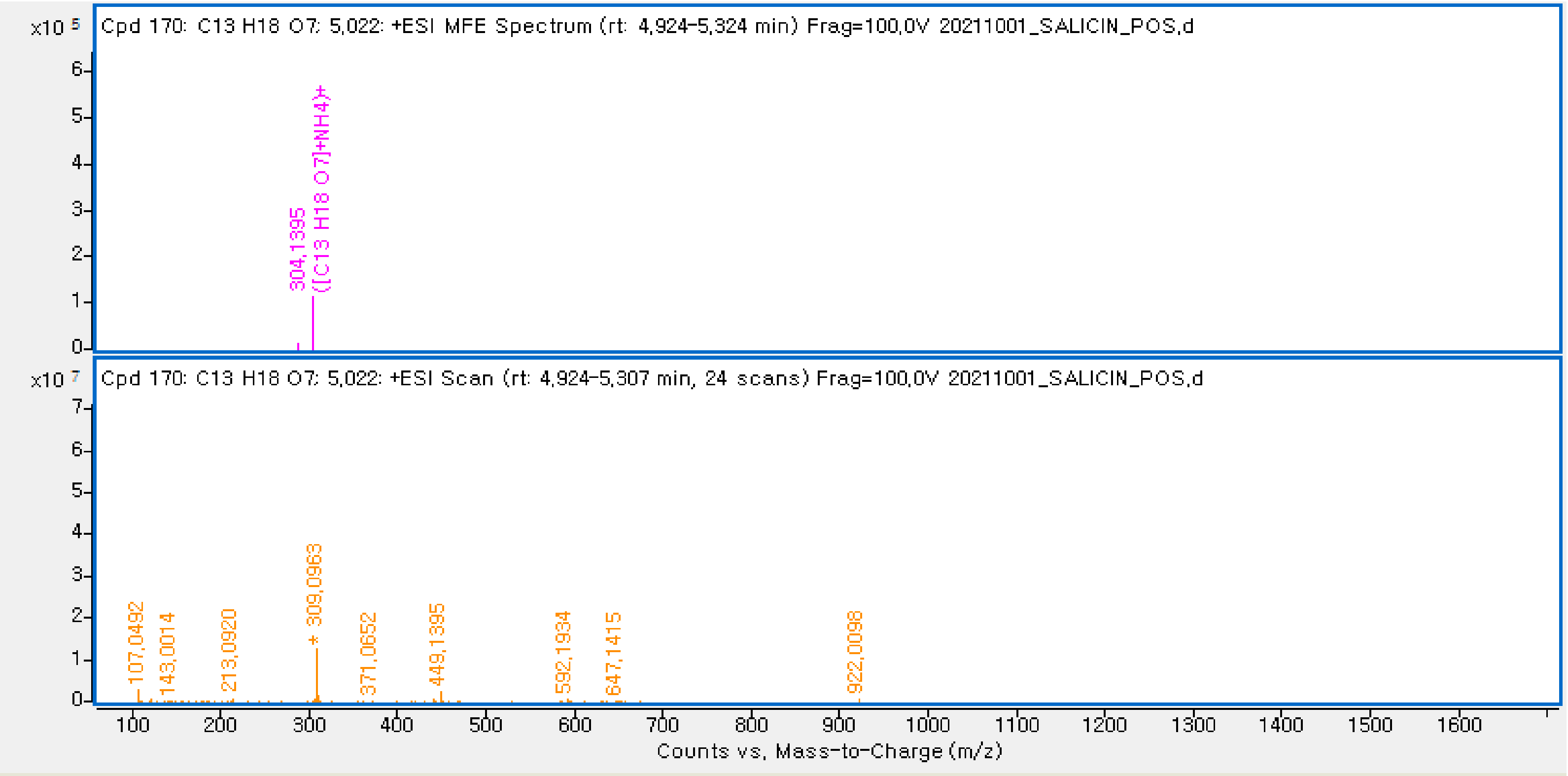

Supplement: Supplementary file 1 [file plants-12-00104-s001.zip › plants-2074772-supplementary.pdf]
